# The Major Cellular Sterol Regulatory Pathway Is Required for Andes Virus Infection

**DOI:** 10.1371/journal.ppat.1003911

**Published:** 2014-02-06

**Authors:** Josiah Petersen, Mary Jane Drake, Emily A. Bruce, Amber M. Riblett, Chukwuka A. Didigu, Craig B. Wilen, Nirav Malani, Frances Male, Fang-Hua Lee, Frederic D. Bushman, Sara Cherry, Robert W. Doms, Paul Bates, Kenneth Briley

**Affiliations:** 1 Department of Microbiology, Perelman School of Medicine, University of Pennsylvania, Philadelphia, Pennsylvania, United States of America; 2 Department of Pathology and Laboratory Medicine, The Children's Hospital of Philadelphia, Philadelphia, Pennsylvania, United States of America; Mount Sinai School of Medicine, United States of America

## Abstract

The *Bunyaviridae* comprise a large family of RNA viruses with worldwide distribution and includes the pathogenic New World hantavirus, Andes virus (ANDV). Host factors needed for hantavirus entry remain largely enigmatic and therapeutics are unavailable. To identify cellular requirements for ANDV infection, we performed two parallel genetic screens. Analysis of a large library of insertionally mutagenized human haploid cells and a siRNA genomic screen converged on components (SREBP-2, SCAP, S1P and S2P) of the sterol regulatory pathway as critically important for infection by ANDV. The significance of this pathway was confirmed using functionally deficient cells, TALEN-mediated gene disruption, RNA interference and pharmacologic inhibition. Disruption of sterol regulatory complex function impaired ANDV internalization without affecting virus binding. Pharmacologic manipulation of cholesterol levels demonstrated that ANDV entry is sensitive to changes in cellular cholesterol and raises the possibility that clinically approved regulators of sterol synthesis may prove useful for combating ANDV infection.

## Author Summary

As obligate, intracellular parasites viruses are dependent upon the host cell for numerous factors and processes. However, for many important viruses few of the required host factors have been identified. Hantaviruses are rodent-borne viruses that are associated with severe human disease. Transmission to humans occurs sporadically with a recent notable example in Yosemite National park. In the present study, we utilized two independent genetic strategies to discover cellular factors needed for replication of the highly pathogenic hantavirus Andes virus. We found that four genes, encoding components of a complex involved in regulation of cholesterol synthesis and uptake, were critical for Andes virus infection. Drugs that inhibit an enzyme in this complex or that reduce cellular cholesterol levels effectively blocked Andes virus infection, suggesting new ways for combating this pathogenic virus.

## Introduction

Hantaviruses are a genera of the *Bunyaviridae* family that includes a large number of human pathogens. Hantaviruses found in the Americas, the so called New World hantaviruses, including Andes virus (ANDV) from Argentina and Chile, can cause a lethal hemorrhagic fever known as hantavirus pulmonary syndrome (HPS) while the Old World hantaviruses from Europe and Asia are associated with Hemorrhagic Fever with Renal Syndrome (HFRS) [1]–[5]. Unlike other members of the *Bunyaviridae* family, ANDV and the other hantaviruses are not transmitted by arthropod vectors but instead infect humans directly by aerosolized excreta from infected rodents. Entry into host cells by the membrane enveloped hantaviruses depends upon the viral glycoproteins G_N_ and G_C_, which form a heterodimeric complex on the virion surface following cleavage of a polyprotein precursor [6]–[8]. Although it is clear that hantaviral infection relies upon transit to an acidic intracellular compartment where the viral glycoproteins mediate membrane fusion [9], [10], the overall entry process is not fully elucidated.

As with other viruses, ANDV must utilize host cell molecules and pathways during the virus life cycle for replication to occur. However relatively little is known about how ANDV, or other hantaviruses, interact with their host cells. High-throughput genetic screens have changed the way viral host co-factors are identified since these approaches have the ability to reveal not only host cell molecules that directly interact with viral components to facilitate virus infection, but also the cellular pathways that orchestrate the expression and activity of these molecules. Identifying pathways rather than individual molecules that are needed for virus replication could lead to the development of multiple therapeutic targets. Moreover, pathways used in common by multiple viruses within a family would represent ideal candidates for therapeutic development.

To identify cellular factors and pathways important for hantavirus replication, we employed two genetic screens: a haploid human cell line that was insertionally mutagenized with a gene-trap vector and a large-scale siRNA screen. A recombinant vesicular stomatitis virus (VSV) recombinant in which the ANDV glycoproteins are expressed on a VSV core (rVSV-ANDV [11]) focused our screening efforts on cellular processes involved in early steps of the ANDV infectious pathway. Key findings were confirmed with replication competent, wild-type ANDV. These independent genetic screens identified members of the major cellular cholesterol regulatory pathway as important for ANDV entry. Inhibiting this pathway using complementary genetic and pharmacologic approaches demonstrated that ANDV is exquisitely sensitive to the cellular levels of cholesterol. Decreased cellular cholesterol blocked ANDV infection at the level of virus entry. Despite normal binding to the cell surface, virus failed to be internalized, resulting in a profound block to infection. Overall these studies provide a framework with which to identify additional cellular components involved in the entry of ANDV, and potentially other hantaviruses, and raise the possibility that approved inhibitors of sterol regulation and synthesis may find clinical application in treating ANDV infection.

## Results

### Independent genetic screens identify members of the cholesterol regulatory complex as required for the entry of recombinant ANDV

**Figure 1:**
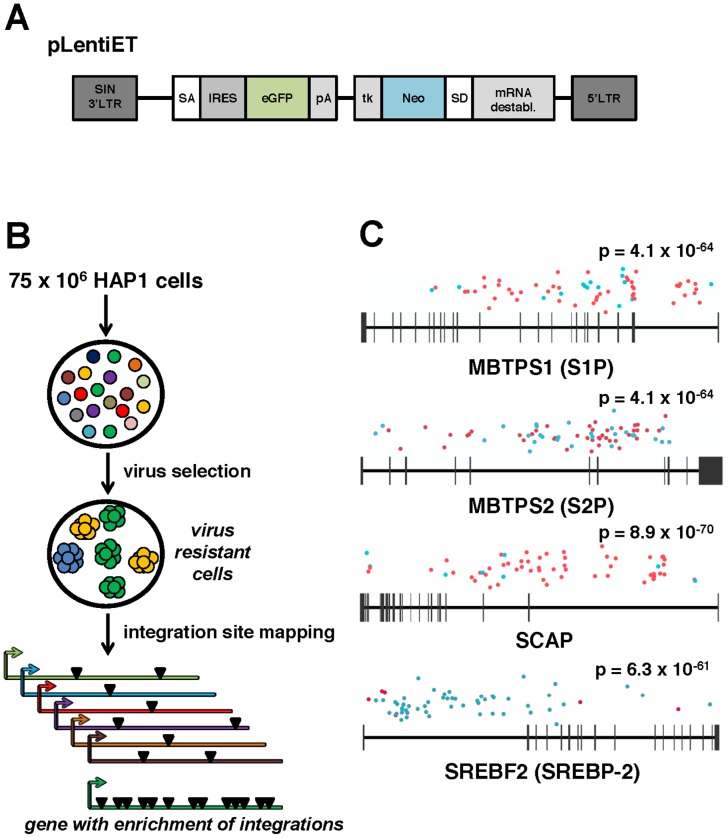
A forward genetic screen in human haploid cells identifies MBTPS1, MBTPS2, SCAP, and SREBF2 as required for ANDV infection. (A) pLentiET gene-trap vector schematic. pLentiET is diagramed 3′ to 5′, self-inactivating (SIN) 3′LTR, splice acceptor site (SA), internal ribosome entry site (IRES), eGFP, polyadenylation signal (pA), tk promoter (tk), G418 resistance gene (Neo), splice donor site (SD), mRNA destabilization element, 5′ LTR. Upon a *successful* gene-trapping event, eGFP is spiced into the coding transcript downstream of the native promoter. A second transcript encoding G418 is simultaneously produced. (B) Genetic screen strategy. 75×10^6^ human haploid (HAP1) cells were mutagenized using a gene-trap vector (pLentiET) delivered through transduction. Cells were expanded for several passages and infected with either rVSV-ANDV or rVSV-G virus to select for cells resistant to infection. Resistant cells were used for gene-trap integration site analysis (total chromosomal DNA from all resistant cells), integrations aligned to the human genome, and integration frequency ranked. (C) MBTPS1 (Site 1 Protease, S1P), MBTPS2 (Site 2 Protease, S2P), SCAP (Sterol Regulatory Element Binding Protein Cleavage Activating Protein), and SREBF2 (Sterol Regulatory Element Binding Protein 2, SREBP-2) were found to have the highest total number of independent integration events. Genes are depicted 5′ to 3′ with horizontal bars denoting exons. Sequenced integration sites are demarked based on the orientation of the gene-trap vector relative to the human genome (red dots: sense strand, blue dots: antisense stand). Significance values were calculated using the 1-sided Fisher's Exact test based on the unselected control library are indicated above each gene.

As one approach to identify human genes required for ANDV entry we employed an insertional mutagenesis strategy (Figure 1B) in the human haploid cell line (HAP1, [12]). Approximately one billion HAP1 cells were transduced with a gene-trapping vector, LentiET (Lentiviral Exon Trap, Figure 1A), to generate a library of cells with insertionally-inactivated genes. Survival of parental and mutagenized HAP1 cells was selected for in parallel with either replication competent recombinant Vesicular Stomatitis Virus that uses its endogenous glycoprotein (rVSV-G) or a replication competent VSV enveloped with the glycoprotein of ANDV (rVSV-ANDV). Challenge of ∼75 million LentiET mutagenized HAP1 cells with rVSV-ANDV produced hundreds of colonies while parental HAP1 cells yielded no surviving colonies when infected with rVSV-G or rVSV-ANDV. In contrast to the results with rVSV-ANDV, no mutagenized HAP1 cells survived selection with rVSV-G. Since the mutagenized cells specifically survived rVSV-ANDV infection, the infection resistance maps to the ANDV glycoprotein and not to the replication of the VSV core.

Mutagenized HAP1 cells surviving rVSV-ANDV selection were pooled and an aliquot was tested for sensitivity to rVSV-ANDV and rVSV-G infection. Confirming the results of the screen, the selected population displayed significant resistance to rVSV-ANDV infection while remaining sensitive to rVSV-G (Figure S1). Genomic DNA prepared from pooled rVSV-ANDV-resistant cells was used to map pLentiET genetic integration sites. In total, 676 independent integrations sites were mapped in the human genome with 80% being intragenic (Table S1). Of these insertions, 253 (37%) were located within four genes of a sterol regulatory element-binding protein pathway: Sterol Regulatory Element Binding Protein 2 (*SREBF2*; 59 insertions), Sterol Regulatory Element Binding Protein Cleavage Activating Protein (*SCAP*; 62 insertions), Site 1 Protease (*S1P*; 62 insertions), and Site 2 Protease (*S2P*; 70 insertions), (Figure 1C). These insertional frequencies were compared to the insertional frequency within the HAP1 library pre-virus selection. As the p-values indicate (Figure 1C, all less than 1×10^−60^), it is highly unlikely for these genes to have been enriched by chance. Furthermore, no other genes contained more than 2 integrations within the selected population (Table S1). In addition, integrations within SCAP and S1P highly favor the orientation where the gene trap vector effectively “captures” the transcript whereas the opposite is true for the SREBF2 gene (Figure 1C, Table S1).

In parallel an RNAi screen was performed using an optimized high throughput luciferase-based assay in a human HEK293T cell line engineered to constitutively express Firefly Luciferase (ffLuc). The Ambion Druggable Genome library (9,102 genes) was employed in a 384-well format with 4 siRNAs per gene and 2 siRNAs per well. Infection was assessed using a non-replicating ANDV (VSV-(ANDV)) viral pseudotype system in which the native glycoprotein (VSV-(G)) was replaced with a Renilla Luciferase (rLuc) reporter and the ANDV glycoprotein was provided *in trans* [13]. This assay exhibited a linear correlation between rLuc activity and the amount of input virus across a 4-log titration (data not shown). Likewise, the ffLuc signal derived from the HEK293T cell line correlated with cell number across a broad range and so could be used to assess cell viability in each well (Figure S2). The library was reverse-transfected into the HEK293T-ffLuc cells and 72 hours post-transfection cells were infected with VSV-(ANDV). Twenty-four hours post-infection, relative light units (RLU) for cell viability (firefly) and infection (Renilla) were measured. For each plate, robust Z scores were calculated for both cell viability and infection. Genes were identified as hits with a robust Z score for infection of <−1.5 in both siRNA pools (p<0.009). This stringent approach requires that at least 2 unique siRNAs against the gene of interest impacted infection. siRNAs were considered cytotoxic and excluded if they had a robust Z score for viability <−2 in both pools. Based on these criteria, 105 genes were identified as important for infection (Table S2A).

To validate the genes identified in the RNAi screen and to differentiate genes important for ANDV glycoprotein-mediated entry from those related to replication of the VSV core, 3 additional, unique siRNAs targeting 96 of the initial hits were screened using both ANDV and VSV-(G) viral pseudotypes (Table S2A). Genes validated if at least one additional siRNA inhibited infection in at least two biological replicates of the secondary screen, using a cut-off of a robust Z score for infection of <−1.3 (p<0.05) with no cytotoxicity (robust Z score>−2). Thirty-three genes met these criteria, with 9 specific for ANDV glycoprotein-mediated entry (Table 1, Table S2B). Comparison of these results with the haploid cell screen revealed that *SREBF2* was the only gene in common, making this a strong candidate since it influenced infection in two different screens.

**Table 1:** List of candidate genes identified during siRNA screen.

	Primary Screen		Secondary Screen	
Gene	ANDV		ANDV	VSV
	Infection	Cell Viability	Infection	Infection
**SREBF2**	−2.059	−0.647	−1.736	−0.643
CES2	−2.162	−1.823	−2.104	−0.912
HSPA2	−1.780	−1.021	−2.135	−0.781
IQGAP3	−2.341	−2.164	−1.828	−0.645
LGR6	−2.022	−0.713	−1.511	−0.756
MCAT	−1.952	0.421	−1.810	−1.440
NAP1L4	−2.065	0.965	−1.694	−1.165
PTGS2	−1.907	−0.090	−1.983	0.027
ST3GAL2	−2.392	−0.385	−2.711	−1.127

List of validated genes identified in the siRNA screen. Shown are averaged robust z-scores for the primary and secondary screens. Genes were identified in the primary screen as having a robust z-score for infection <−1.5 in both wells; robust z-score<−2 for cell viability in both wells was criteria for exclusion. Genes validated in the secondary screen with robust z-score<−1.3 for infection normalized to cell viability in duplicate wells in ≥2 biological replicates for ANDV, but not for VSV.

### SREBP-2, SCAP, S1P, and S2P are required for entry of rVSV-ANDV

**Figure 2:**
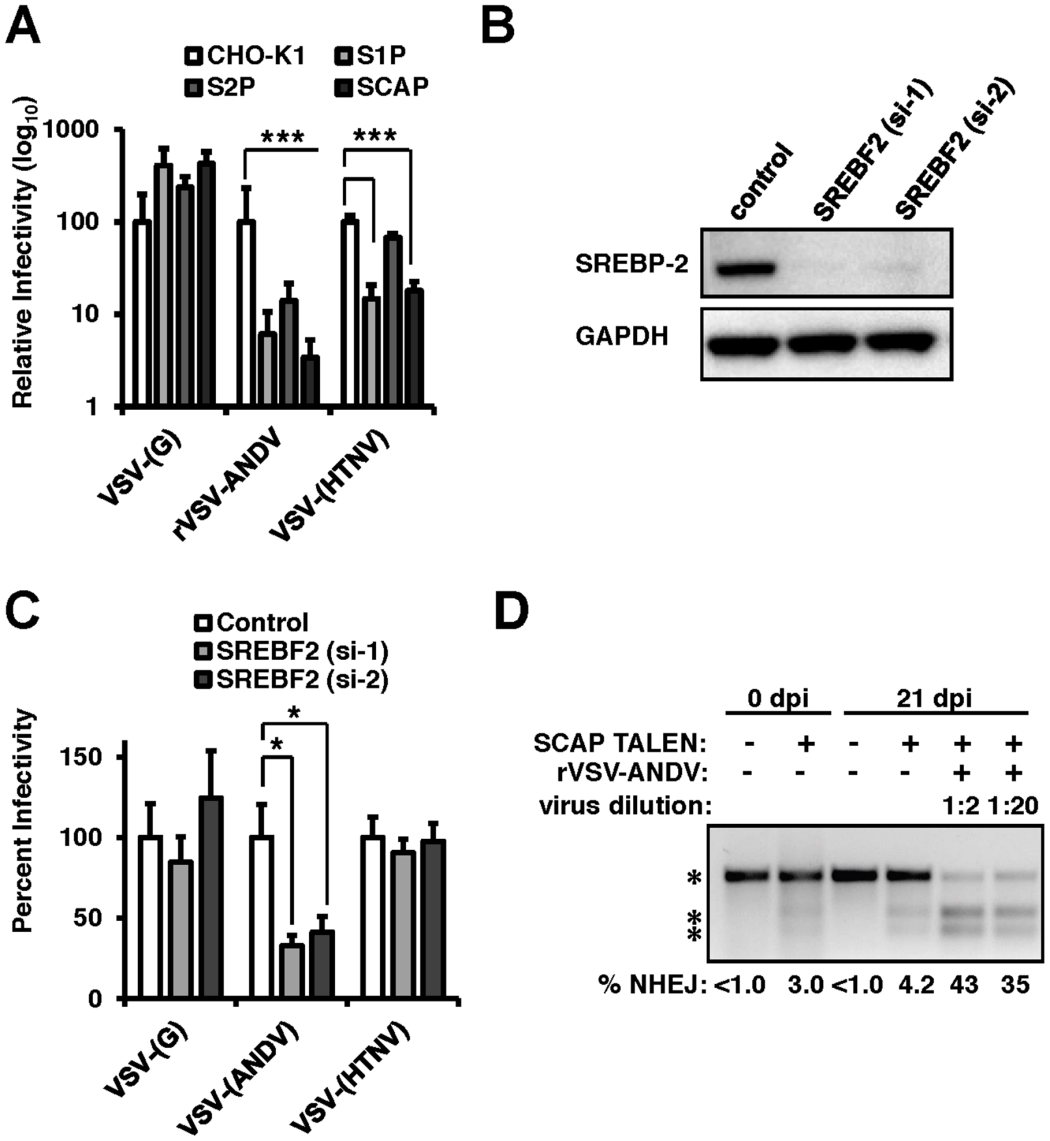
The sterol regulatory complex promotes infection of ANDV. (A) Infection of Chinese Hamster Ovary (CHO) cells null for S1P, S2P, or SCAP (Figure S2). CHO cells were infected with VSV-(G), rVSV-ANDV or rVSV-HTNV for 10 hours. Infections were quantified via flow cytometry and normalized relative to wild-type (CHO-K1) cells. Mean±SEM shown for three independent experiments; *** p<0.001. (Raw infection percentage (CHO-K1): VSV-(G) = 21%, rVSV-ANDV = 15%, VSV-(HTNV) = 8%) (B) siRNAs directed against SREBF2 efficiently knock down protein expression as measured by immunoblot to SREBP-2 with GAPDH as a loading control. (C) HEK293T cells depleted of SREBF2 were infected with non-replicating VSV pseudotypes bearing the indicated glycoproteins encoding red fluorescent protein (RFP) and infection was quantified 10 h.p.i.. Infectivity was normalized relative to control cells. Mean±SEM shown for three independent experiments; * p<0.05. (Raw infection percentage (control): VSV-(G) = 4%, VSV-(ANDV) = 19%, VSV-(HTNV) = 3%) (D) TALENs were used to genetically disrupt SCAP in HEK293T cells. These insertions were enriched upon challenge with rVSV-ANDV and were all killed by VSV-G. Enrichment was measured by a non-homologous end joining (NHEJ) endonuclease assay where a single band (top asterisk) indicates the presence of wild-type sequence and the lower molecular weight doublet (lower 2 asterisks) represents the disrupted alleles. Densitometry was used to quantify the levels of the undisrupted and disrupted alleles, which is shown below.

The results obtained from the siRNA and haploid cell screens indicated that components of the sterol regulatory pathway (*SREBF2, SCAP, S1P*, and *S2P*) were required for rVSV-ANDV infection. To probe the importance of this pathway for recombinant ANDV infection, a panel of well-characterized Chinese Hamster Ovary (CHO) cell lines individually null for S1P, S2P, or SCAP (Figure S3) [14]–[16] were challenged with VSV pseudotypes bearing the VSV or ANDV glycoproteins (Figure 2A). Additionally, these cell lines were infected with VSV pseudotypes carrying the glycoproteins from an Old World hantavirus, Hantaan virus (HTNV). rVSV-ANDV infection was severely impaired showing a 1–2 log decrease in each of the mutant CHO cell lines, whereas the infection level by viral pseudotypes bearing the VSV-(G) glycoprotein was similar to the parental CHO cells. Infection with VSV-(HTNV) decreased by roughly 1-log in cells null for S1P and SCAP, but not S2P, suggesting a more modest dependence on this pathway. A stable cell line lacking *SREBF2* was not available, therefore SREBP-2 expression was knocked-down with two independent siRNAs in HEK293T cells (Figure 2B). Knockdown of SREBP-2 expression resulted in a significant (p<0.05) decrease in VSV-(ANDV) infection with no significant impact on VSV-G or VSV-(HTNV) infection (Figure 2C).

To explore the importance of the cholesterol regulatory complex for ANDV glycoprotein-dependent infection in human cells we developed a Transcription Activator Like Exonuclease (TALEN) pair that disrupted the coding region of *SCAP* (TALEN_SCAP_). HEK293T cells transfected with TALEN_SCAP_ were expanded and infected with rVSV-ANDV to kill susceptible cells (Figure 2D). *SCAP* disruption was quantified pre- and post-infection using a quantitative PCR heteroduplex cleavage assay [17]. The heteroduplex assay revealed that following TALEN_SCAP_ transfection, ∼3% of the population had evidence of gene disruption. After infection with rVSV-ANDV, the SCAP-disrupted population increased to ∼43%. Sequence analysis confirmed the gene disruption (Figure S4). Enrichment for the disrupted *SCAP* gene was not observed in cells passaged without virus infection (Figure 2D). Wild-type VSV efficiently infected and killed the SCAP disrupted cells (data not shown). Taken together, these three independent experimental approaches provide strong genetic evidence that members of this sterol regulatory complex are required for efficient ANDV glycoprotein-mediated infection in diverse cell types and hosts.

### Cholesterol pathway inhibitors block Andes virus glycoprotein-mediated infectivity

**Figure 3:**
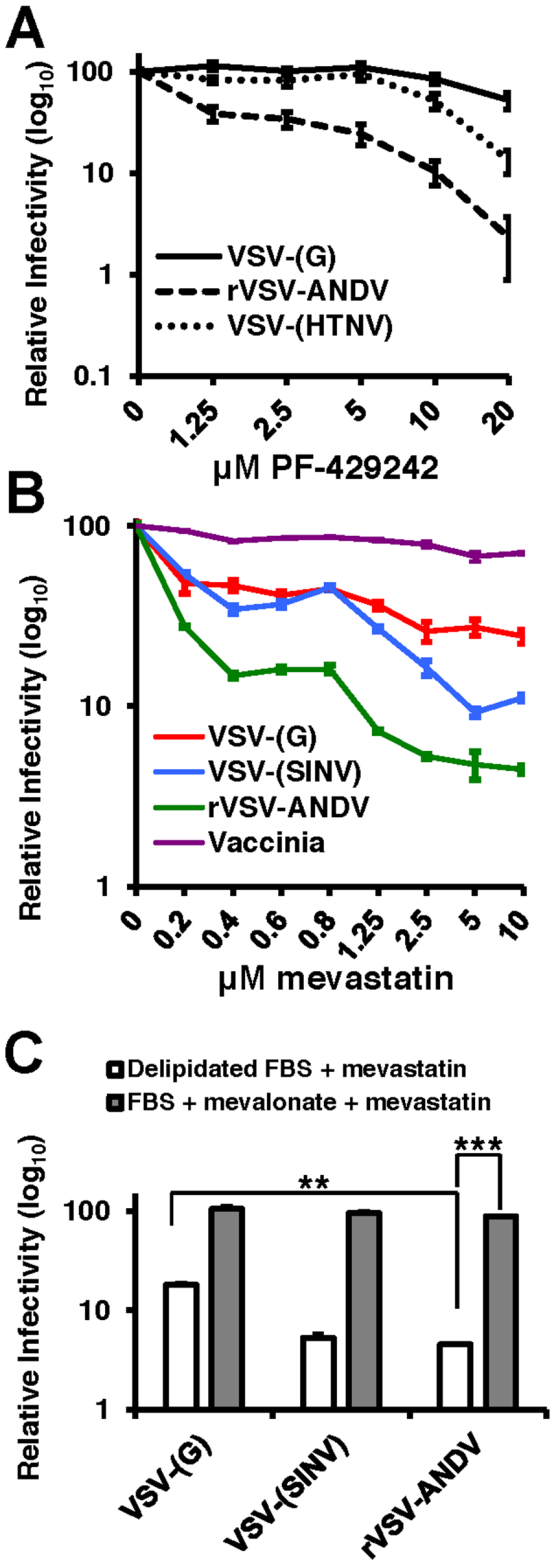
PF-429242 and mevastatin prevent efficient infection by rVSV-ANDV. (A) A549 cells were pretreated with the indicated concentrations of PF-429242 24 hours prior to infection with VSV-(G) (solid line), rVSV-ANDV (dashed line) or VSV-(HTNV) (dotted line). Cells were harvested 16 hours later and viral infection levels were quantified via flow cytometry. Values are normalized to untreated infection levels for each virus. Mean±SEM shown for three independent experiments; p<0.001 for 20 µM drug concentration for both rVSV-ANDV and VSV-(HTNV) compared to untreated control. (Average raw infection percentage (untreated): VSV-(G) = 45%, rVSV-ANDV = 35%, VSV-(HTNV) = 14%). (B) Treatment of A549 cells with the HMG-CoA Reductase inhibitor, mevastatin, blocks infections with Sindbis (SINV) and ANDV pseudoviruses. VSV-derived pseudoviruses (VSV-(G) and VSV-(SINV)), rVSV-ANDV or vaccinia virus were used to infect cells pretreated with the indicated concentrations of mevastatin. Infections were quantified using flow cytometry either by fluorescent protein expression or indirect antibody staining (rVSV-ANDV). Infectivity is normalized relative to untreated control cells. Mean±SEM shown for three independent experiments; p<0.001 for 10 µM drug concentration for rVSV-ANDV compared to untreated control. (Average raw infection percentage (untreated): VSV-(G) = 33%, VSV-(SINV) = 16%, rVSV-ANDV = 52%, vaccinia = 30%). (C) Viral infectivity in the presence of 5 µM mevastatin can be rescued with the addition of normal FBS and mevalonate. A549 cells were treated as described above with DMSO or 5 µM mevastatin with delipidated FBS or normal FBS (not subjected to delipidation) with 25 µM mevalonate. Cells were infected with pseudoviruses (VSV-(G)or VSV-(SINV)) or rVSV-ANDV encoding RFP and harvested 10 h.p.i. Infections were quantified using flow cytometry. Infectivity has been normalized relative to untreated control cells. Mean±SEM is shown for three independent experiments; ** p<0.01, ***p<0.001. (Average raw infection percentage (FBS+DMSO): VSV-(G) = 49%, VSV-(SINV) = 14%, rVSV-ANDV = 45%).

PF-429242 is a reversible, competitive inhibitor of S1P that blocks cleavage and subsequent of SREBP-2 and has been shown to reduce the rates of cholesterol synthesis in cultured cells and in mice [18]. Human airway epithelia derived A549 cells were pretreated with varying concentrations of PF-429242 for 24 hours to allow turnover of activated SREBP-2 and subsequently infected with VSV-(G), rVSV-ANDV or VSV-(HTNV) (Figure 3A). As expected, treatment with PF-429242 caused a dose-dependent reduction in the levels of total cellular cholesterol (Figure S5). rVSV-ANDV infectivity also decreased in a dose-dependent manner with an approximately 50-fold reduction at 20 µM PF-429242 (dashed line). PF-429242 had an intermediate effect on the infectivity of VSV-(HTNV) (dotted line). In contrast, VSV-(G) infection of A549 cells is only modestly inhibited in the presence of PF-429242 (Figure 3A; continuous line) at this concentration (20 µM), however it is increasingly abrogated at concentrations ≥40 µM (data not shown). Inhibition of rVSV-ANDV by PF-429242 was not due to delayed viral entry kinetics since the vast majority of fusion had occurred within the first 3 hours in the presence or absence of PF-429242 (Figure S6).

To address whether statins, a clinically approved class of cholesterol lowering drugs, could significantly reduce rVSV-ANDV infectivity, we selectively inhibited cholesterol synthesis with the HMG-CoA reductase inhibitor mevastatin 24 hours prior to infection in delipidated growth medium. Pretreatment of human A549 cells with 1.25 µM mevastatin reduced rVSV-ANDV infectivity (>10-fold, Figure 3B). Controls included a VSV-Sindbis virus pseudotype (VSV-(SINV)) known to be sensitive to sterol levels, and vaccinia virus which is not affected by cellular cholesterol [19], [20]. VSV-(SINV) and rVSV-ANDV displayed a dose-dependent effect of mevastatin on infection, with rVSV-ANDV appearing significantly (p<0.05) more sensitive than VSV-(SINV) (Figure 3B). Infection by VSV-(G) also displayed a dose-dependent decrease compared to untreated cells; however the magnitude of this decrease was significantly less pronounced compared to rVSV-ANDV (Figure 3B). As anticipated, infection by a cholesterol-independent virus, vaccinia, was unaffected by mevastatin treatment (Figure 3B). Supplementation of media with mevalonate and sera reversed the inhibitory effect of mevastatin on rVSV-ANDV infection (Figure 3C). This complementation was likely the result of a combination of mevalonate uptake and LDL scavenging from this rich FBS, since it is expected that the inhibition of cholesterol synthesis would enhance expression of the LDL receptor. The increase in rVSV-ANDV infection under these conditions is consistent with the observation that cholesterol levels in statin-treated cells grown in FBS supplemented with mevalonate rebound to near wild-type levels (data not shown). Overall, VSV-G-dependent infectivity is largely unaffected (<2-fold) by 20 µM PF-429242, or in the loss of SCAP, S1P, or S2P, or depleted SREBP-2, yet mevastatin does appear to have a greater impact on VSV-(G) infectivity. This effect may be the result of LDL-R surface expression dynamics as this family of proteins have been shown to act as VSV-(G) receptors [21].

### Wild-type Andes virus infection is sensitive to cholesterol depletion

**Figure 4:**
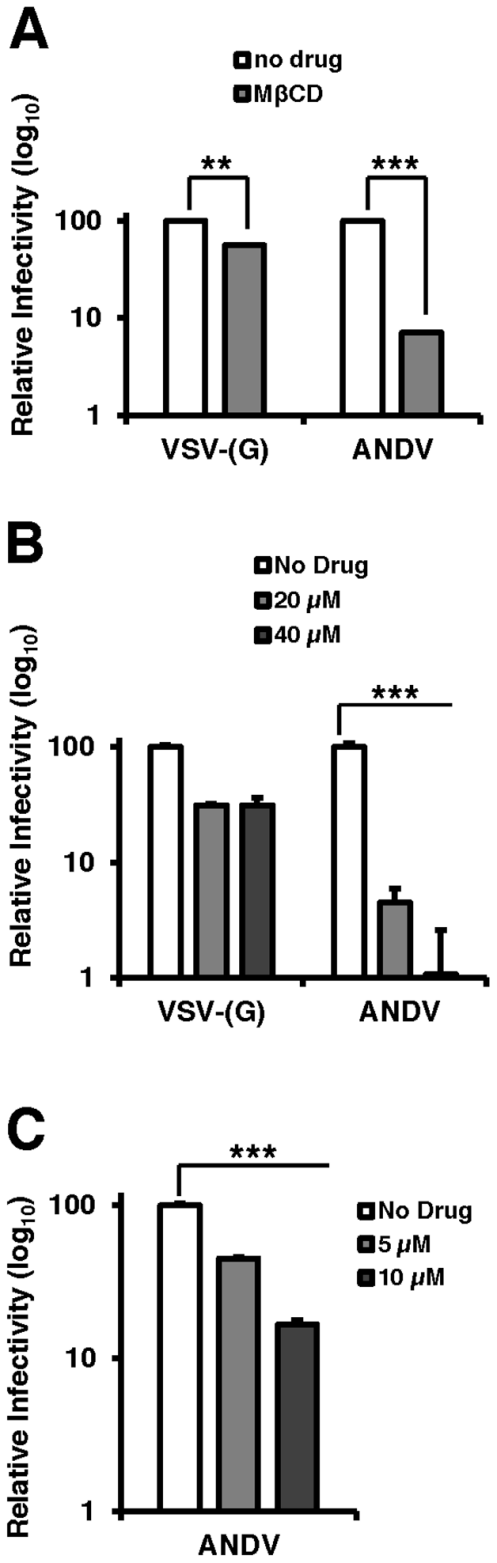
Cholesterol is required for ANDV infection. Infection of cells pretreated with (A) PF-429242 or (B and C) mevastatin blocks infection with wild-type ANDV (strain 9717869). Vero E6 cells were pretreated with DMSO, PF-429242, or mevastatin at the indicated concentrations and 24 hours later, cells were infected with virus at an MOI of 3 for 2 hours and subsequently overlaid with fresh media. All infections and incubations were carried out in the presence of indicated concentrations of PF-429242 or mevastatin. 72 h.p.i (ANDV) or 16 h.p.i. (VSV-(G)) cells were harvested for flow cytometry, and the percentage of infected cells was determined by expression of a fluorescent reporter gene (VSV) or indirect fluorescent staining of ANDV N (ANDV). Values are normalized to infection rate of DMSO treated cells. Mean±SEM shown for three independent experiments; ** p<0.01, ***p<0.001. (Average raw infection percentage of PF-429242 experiments (No Drug): VSV-(G) = 53%, ANDV = 9% and mevastatin experiments (No Drug): ANDV = 16%).

Collectively, the data presented thus far have established that the cholesterol regulatory pathway leading to SREBP-2 cleavage is required for infection by viruses bearing the ANDV glycoproteins. Because this pathway regulates a number of genes required for sterol biosynthesis and internalization as well as cholesterol production within the cell, we wished to investigate whether cellular cholesterol levels are important for ANDV glycoprotein mediated infection. To this end, cells were treated with methyl-β-cyclodextrin (MβCD), which extracts sterols from membranes [22]. Additionally, wild-type ANDV (strain 9717869) was employed for this analysis. MβCD treatment of Vero cells had a modest effect (2-fold) on infection mediated by VSV-(G), whereas ANDV infection was inhibited by more than 10-fold (Figure 4A). Treatment with MβCD reduced total cellular cholesterol levels to ∼80% of untreated samples (data not shown).

To determine if the cholesterol depleting drugs used above to effectively block rVSV-ANDV could also inhibit infection by wild-type ANDV, Vero E6 cells pretreated with PF-429242, mevastatin, or DMSO were infected with ANDV. Three days post infection, cells were fixed, labeled with antibodies against the ANDV nucleoprotein (ANDV-N), and infectivity was determined by flow cytometry (Figures 4B and C). Treatment with either PF-429242 or mevastatin had a significant and dose-dependent effect on ANDV infection, decreasing the percentage of infected cells by approximately 100-fold at 40 µM PF-429242 (Figure 4B) and 6-fold at 10 µM mevastatin (Figure 4C). In contrast, treatment with PF-429242 diminished the infectivity of VSV-(G) with roughly a 3-fold effect at both 20 µM and 40 µM concentrations (Figure 4B). Overall, the MβCD, PF-429242, and statin experiments provide strong evidence that cellular cholesterol levels dictate the permissivity of cells to ANDV infection.

### The activity of S1P is required for virus internalization

**Figure 5:**
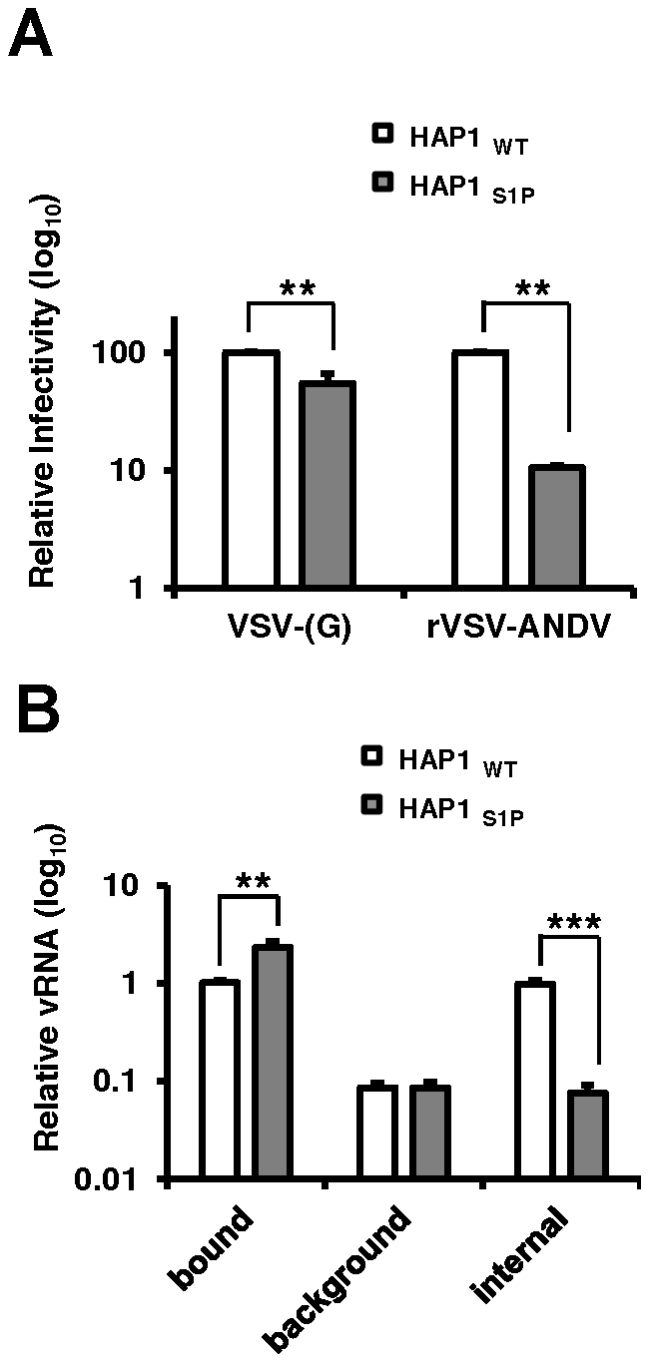
Quantitative PCR analysis of viral RNA during infection of human cells. (A) HAP1_WT_ or HAP1_S1P_ cells were infected with VSV-(G) or rVSV-ANDV for 12 hours and cells collected for flow cytometric analysis. Infection was normalized to infection levels in HAP1_WT_ cells. Mean±SEM is shown for five independent experiments; ** p<0.01. (Average raw infection percentages (HAP1 WT): VSV-(G) = 55%, rVSV-ANDV = 71%) (B) Binding and internalization of rVSV-ANDV. rVSV-ANDV was bound to three sets of HAP1_WT_ and HAP1_S1P_ cells for one hour on ice. One set of cells were scraped into PBS and washed to measure bound virions (bound). A second set of cells were treated with trypsin for 10 minutes to remove externally bound virions (background). A third set of cells were warmed to 37°C for one hour to permit endocytosis before being treated with trypsin to remove any remaining external virions (internal). Cells were washed extensively, cell pellets and associated virions were lysed for RNA extraction and viral RNA (vRNA) was quantified by qRT-PCR. Viral RNA values were normalized to GAPDH to control for input RNA levels and plotted relative to virus bound to HAP1_WT_ cells. Mean±SEM is shown for three independent experiments; ** p<0.01, ***p<0.001.

To investigate the mechanism by which cells lacking a functional cholesterol regulatory pathway resist ANDV infection, we compared early stages of the rVSV-ANDV replication cycle in HAP1 cells with an insertional LentiET mutation (not shown) into the S1P gene (HAP1_S1P_) that abrogates S1P protein expression (Figure S7). HAP1_S1P_ cells were resistant to infection by rVSV-ANDV as compared with rVSV-G (Figure 5A), similar to studies in mutant CHO cells. Next, a qRT-PCR assay was used to monitor binding and internalization of incoming rVSV-ANDV particles. Binding was performed at 4°C with equal amounts of virus added to wild-type or HAP1_S1P_ cells. After extensive washing to remove unbound virus, S1P-deficient cells bound ∼2-fold more rVSV-ANDV virions than wild-type cells (Figure 5B) despite the fact that infection levels were ∼10-fold lower (Figure 5A). As expected for surface bound virus, protease treatment decreased the PCR signal by more than 90% for both cell lines (Figure 5B; *background*). To measure virus uptake, rVSV-ANDV was bound at 4°C, then cells were transferred to 37°C for one hour to allow virus internalization. We chose this time point because we found that rVSV-ANDV is resistant to the lysosomotropic agent ammonium chloride (NH_4_Cl) by one hour post infection, indicating that acid-dependent membrane fusion has occurred by this time (Figure S8). After internalization, cells were treated with protease to remove any remaining external virions (Figure 5B; *internal*). HAP1_WT_ cells internalized nearly 100% of the measured bound virus (Figure 5B; compare HAP1_WT_ *bound* to *internal*). While this appears remarkably efficient, there is precedent for high levels of internalization with other viruses, as 90% of bound influenza virions are internalized under similar conditions [23]. In contrast, S1P-deficient cells were unable to internalize rVSV-ANDV virions (Figure 5B; compare HAP1_WT_ to HAP1_S1P_ *internal*). Indeed, the amount of viral RNA inside these cells was comparable to the background levels of virus detected on the surface of cells stripped with protease prior to endocytosis (Figure 5B; compare HAP1_S1P_ *internal* to HAP1_WT_ and HAP1_S1P_ *background*). Given that cells lacking S1P possess a ten-fold defect in both ANDV-glycoprotein mediated internalization (Figure 5B) and infectivity (Figure 5A), we infer that this internalization defect is responsible for the resistance.

**Figure 6:**
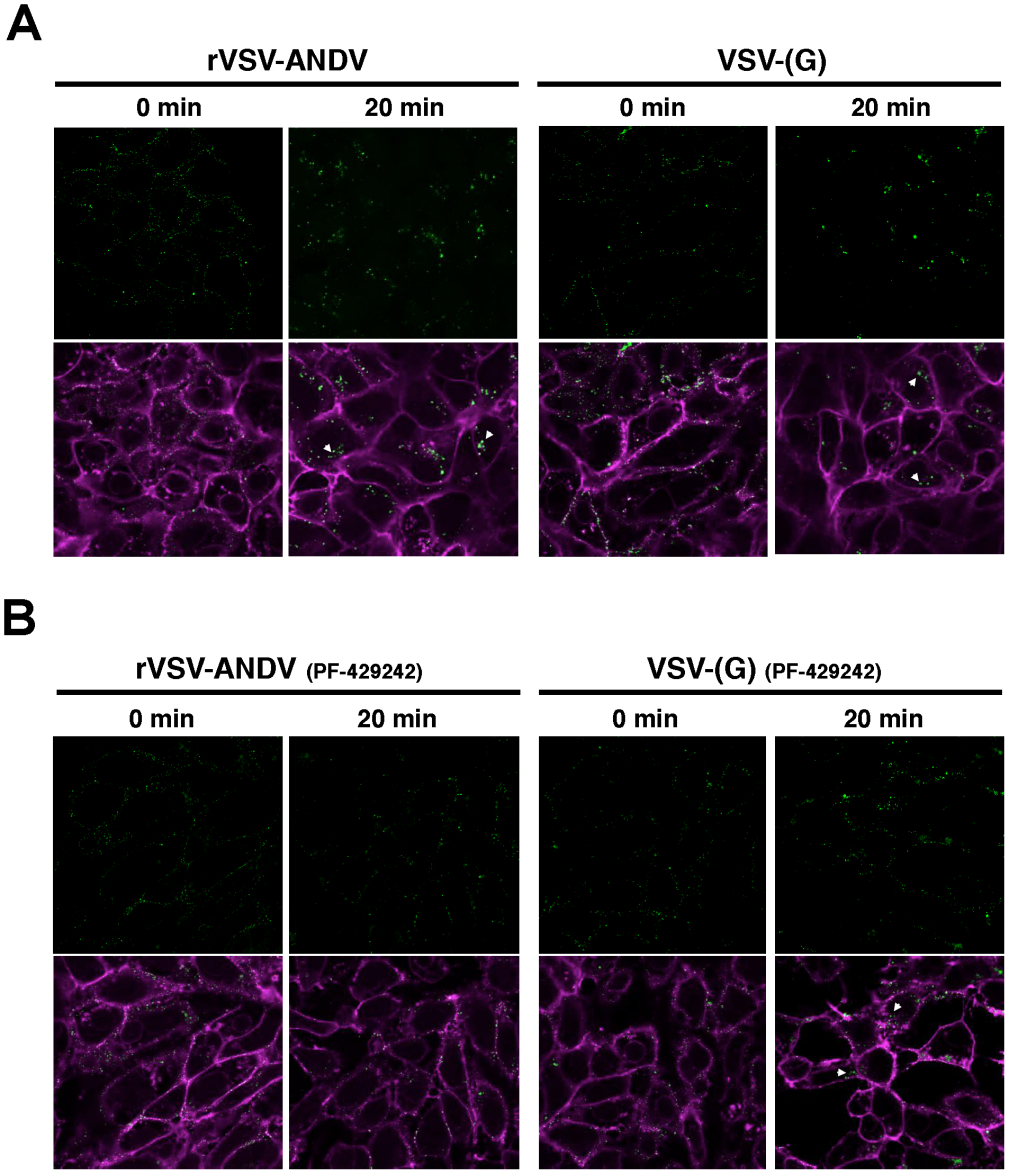
Detection of viral particles during cellular entry. Purified and DiO-labeled rVSV-ANDV (left) or VSV-(G) (right) where bound to Vero E6 cells at 4°C for 90 minutes pretreated for 24 hours with DMSO (A) or 40 µM PF-429242 (B). Cells were subsequently washed with cold PBS and fixed immediately (‘0 min’), or following a 20 minute incubation at 37°C, with paraformaldehyde. Cellular membranes where counterstained Wheat Germ Agglutinan-647 and imaged using confocal microscopy. Internalized virus is indicated with an arrowhead. Representative images from 2 independent experiments are shown.

To investigate this further, we performed confocal microscopy on Vero E6 cells infected with DiO-labeled viral particles (Figure 6). This allows us to track incoming particles. Furthermore, DiO will stain endocytic compartments following viral fusion so we can monitor binding, uptake and fusion events [24]. Vero E6 cells were pretreated with DMSO (Figure 6A) or the S1P-inhibitor PF-429242 (40 µM, Figure 6B) for 24 hours. Following treatment, cells were chilled to 4°C and incubated for 90 minutes with sucrose-purified viral particles previously stained with the lipophilic dye DiO. As a control for the virion preparation, DiO-labeled concentrated and purified supernatant from mock-infected cells did not produce visible puncta on cells (Figure S9). Following incubation, cells were extensively washed with cold PBS and fixed with paraformaldehyde immediately (‘0 min’), or following an incubation at 37°C (‘20 min’). Cellular membranes where counterstained with Wheat Germ Agglutinan-647 and imaged by confocal microscopy. In the absence of drug, both rVSV-ANDV (left) and VSV-(G) (right) samples displayed similar patterns of distribution- at 0 minutes puncta are found distributed along the cellular membranes, and appear internalized with larger puncta after 20 minutes. Although viral fusion could have occurred by 20 minutes, DiO will stain endocytic compartments zfollowing viral fusion [24]. In agreement with the qRT-PCR data, pretreatment of cells with PF-429242 appears to restrict DiO-labeled rVSV-ANDV to the cell periphery (Figure 6B, left). Uptake of labeled VSV-(G) also appears to be impaired (Figure 6B, right) and is consistent with the observed 3-fold decrease in VSV-(G)-infectivity of these cells at 40 µM PF-429242. The results are representative of two independent experiments. Although not quantitative, the microscopy results in S1P inhibited cells, coupled with the qPCR analysis in cells carrying a genetic lesion in S1P, suggest that a functional cholesterol regulatory pathway is needed for effective internalization and subsequent infection by ANDV.

## Discussion

Viral entry is a complex process often requiring orchestration of protein-protein interactions, cellular signaling, and cellular uptake mechanisms [25]. To begin dissecting this process for ANDV, two independent genetic screens were performed. In the first, insertional mutagenesis was carried out with a gene-trap vector in human haploid cells, a method used previously to identify host cell molecules and pathways used by an array of viral and bacterial pathogens [12], [26]–[33]. This approach allows near saturation of the human genome, although genes required for cell viability *in vitro* cannot be interrogated. In the second method, a large-scale RNAi screen provided a complementary approach by producing varying degrees of gene suppression and allowing one to potentially query genes required for cell viability. Previously, the low level extent of overlap in genes discovered in various RNAi screens for the same pathogen has hampered identification of specific requirements [34]–[37]. By employing both of these genetic approaches, we sought to identify cellular pathways important for the early stages of ANDV infection comprehensively. The discovery of components of a cholesterol regulatory complex as an ANDV entry requirement by these two independent screens reinforces the significance of this finding. Moreover, the identification of more than 60 independent insertional mutations in each of the 4 genes of this complex attests to the strength of this observation.

Interestingly, integration into the SREBF2 gene appears to be dramatically skewed toward the “antisense” orientation in which the gene trap vector would not efficiently disrupt gene expression. We hypothesize that these integrations likely cripple, but not fully inactive this gene presumably due to the poor fitness of cells completely deficient for SREBP-2.

The requirement for this sterol regulatory complex in ANDV infection was verified by several orthogonal approaches that included analysis of cells deficient in S1P, S2P, or SCAP function, along with generation and study of TALEN-driven deletions in SCAP, and siRNAs to deplete SREBP-2. These experiments confirmed the importance of this pathway for ANDV infection in multiple species and cells types. In addition, tests of a pharmacologic inhibitor of S1P, statins and cholesterol depletion extended these findings to wild-type ANDV. SREBP-2 functions as an initially ER-resident, regulated transcription factor whose activity is controlled by interactions with SCAP and proteolysis by S1P and S2P. After release from the ER, transit to the Golgi, proteolysis and transport to the nucleus, processed SREBP-2 activates transcription via Sterol Responsive Elements (SREs) upstream of genes such as HMG-CoA reductase, squalene synthase and the low density lipoprotein receptor (LDLR) which increases cholesterol production or uptake. Inactivating any of the four genes that were found to be important for ANDV entry blocks SREBP-2 mediated gene activation resulting in lower levels of cholesterol in the absence of an exogenous source. Given the cellular location of the sterol regulatory pathway components in the ER and cis-Golgi, they appear unlikely to play a direct role in ANDV entry. However, reduced expression of one or more genes transcriptionally induced by SREBP-2 or altered cellular cholesterol levels could account for this phenotype. Pharmacologic inhibitors in conjunction with delipidated media made it possible to separate sterol-dependent gene induction from cellular cholesterol levels. When cellular cholesterol levels were reduced by inhibiting cholesterol synthesis with mevastatin or by treating cells with MβCD, sterol-dependent gene expression is induced. Despite this, ANDV infection remained suppressed by more than 10-fold, arguing that it is the lower levels of cellular cholesterol rather than reduced expression of SREBP-2 target genes that is important. Also consistent with this conclusion is the finding that infection could be restored to cholesterol-depleted cells upon addition of exogenous cholesterol. Altogether, these results demonstrate that ANDV entry is particularly sensitive to perturbations of cellular cholesterol levels.

Several viruses have been shown to exhibit some degree of cholesterol dependence for entry into cells [38]–[41]. In some, the effect of cholesterol depletion is direct and highly specific. The clearest example is the alphavirus Semliki Forest virus (SFV), where a mechanistic role has been demonstrated for cholesterol in membrane fusion. In contrast to our findings with ANDV, cholesterol-depleted cells were unaltered in their ability to bind and internalize SFV, but were blocked at the downstream step of membrane fusion, which blocked subsequent virus replication [42], [43]. For other viruses cholesterol depletion inhibits infection indirectly by inhibiting uptake via endocytic pathways. Caveolin-dependent endocytosis is especially sensitive to cholesterol levels and internalization of non-enveloped viruses such as SV-40 is blocked when cholesterol is depleted [44], [45]. In A549 cells at the concentrations of statins we employed to block ANDV entry, SV-40 infection appeared unchanged (data not shown) suggesting that the observed effects are not due to impaired caveolin-mediated entry. Clathrin-mediated endocytosis, a common route of entry into cells by enveloped viruses, is also sensitive to cholesterol depletion using β-cyclodextrin [46]. However, ANDV as well as rVSV-ANDV infection exhibited exquisite sensitivity to cholesterol depletion and disruption of the sterol regulatory pathway under conditions where VSV-(G) mediated infection, a clathrin dependent process, was only marginally affected. Thus it is unlikely that the mechanism underlying reduced ANDV infection is linked to general clathrin-mediated endocytosis. It is possible that a modest reduction of cholesterol levels impacts lipid raft integrity with concomitant effects on localization of proteins needed by ANDV or upon signaling by proteins that partition into these cholesterol-rich domains. For example, signaling by lipid raft localized DAF1 has been found to be critically important for Coxsackie virus entry [47]. Future studies will be required to examine whether lipid rafts or raft-mediated signaling is important for ANDV entry.

Underscoring the importance of cholesterol homeostasis for viral entry is the recent observation that cellular antiviral systems interfere with cholesterol regulation or trafficking (reviewed in [48]). Interferon induced transmembrane proteins (IFITMs) appear to exert their antiviral activity by causing the accumulation of cholesterol in late endosomal compartments thereby blocking infection of a wide variety of viruses that enter through this compartment [49]. Infection by several *Bunyavirdae* family members, including ANDV, is inhibited by IFITM's [50]. Two other recent studies revealed that interferon induced production of a sterol (oxysterol 25 hydroxycholesterol), which is known to be involved in cholesterol homeostasis, can block infection at the point of viral entry [51], [52]. Although likely mechanistically dissimilar, our results coupled with these findings, highlight the importance of cholesterol homeostasis in viral entry and suggest targeting this process for the development of broadly effective antivirals.

We found that ANDV bound equally well to wild-type and S1P-deficient cells, suggesting that the surface levels of the cellular factors to which ANDV glycoproteins bind are not dependent on the sterol regulatory pathway. Whether ANDV has an absolute requirement for a specific cell surface receptor is not known, though the integrin α_v_β_3_ has been implicated as a binding factor in some cell types [53]–[56]. However, α_v_β_3_ levels were below the limits of detection in the HAP1 cells used for the insertional mutagenesis screen and integrins were not identified in the HEK293-based RNAi screen. Additionally, surface expression levels of α_v_β_3_ on Vero cells were not affected by pharmacologic treatments that blocked ANDV glycoprotein-mediated infection (data not shown). Taken together these observations suggest that α_v_β_3_ integrin is not involved in the cholesterol-dependent phenotype observed in the diverse cells used in these studies. This does not preclude integrin involvement in other cell types.

Although the current screens converged on genes regulating sterol synthesis, it is likely that altering the parameters of the screens by including cholesterol enriched media or constitutively expressing activated SREBP-2 during screening, will uncover additional host factors and/or pathways important for ANDV entry. Finally, the sensitivity of ANDV to safe, effective cholesterol-lowering drugs may suggests new treatments for ANDV infection and pathogenesis.

## Materials and Methods

### Viruses

pLentiET viral pseudotypes were prepared via co-transfection of HEK293T cells with pCAGGS-VSV-(G) (Addgene), pSPAX (Addgene), and pLentiET genome plasmids. Viral supernatants were harvested 48 hours later. Replication competent rVSV-G and rVSV-ANDV were previously described [11], [57]. VSV-(HTNV), VSV-(ANDV), VSV-(SINV) and VSV-(G) pseudovirions were created via coexpression of a VSV-ΔG-*reporter* genome along with a pCAGGS-viral glycoprotein expression plasmid, as previously described [58]. Wild-type ANDV (Chilean strain 9717869) was provided by Connie Schmaljohn at the U.S. Army Medical Research Institute of Infectious Diseases and used under BLS3 conditions. A recombinant Vaccinia virus expressing GFP was previously described [59].

### Haploid cell enrichment, generation and selection of the HAP1 library

HAP1 cells were assessed by fluorescent cytometry for ploidy by Hoechst 33342 staining of nuclei. Haploid cells were enriched by size selection to ∼80% haploid immediately before creation of an insertionally-mutagenized library of ∼1×10^9^ cells using three rounds of mutagenesis with pLentiET gene-trap virus. Virus was added such that ∼80% of cells were transduced per round of mutagenesis as determined by a lenti-GFP control virus made in parallel. ∼75 million library cells were selected with either rVSV-G (MOI of 2) or rVSV-ANDV (MOI of 3–5). Cells selected for resistance to rVSV-ANDV were collected and pooled within 3 weeks and saved as DMSO frozen stocks or used for chromosomal DNA preparation. Clonal populations of HAP1 cells were achieved by limiting dilution.

### Integration site mapping

Chromosomal DNA was prepared from either pools or clonal populations of ANDV resistant HAP1 cells. A DNA amplicon preparation protocol that specifically amplifies LTR-host junctions was carried out essentially as previously described [60]. Amplicons derived from pools of ANDV resistant cells or the unselcted library were subjected to deep sequencing analysis using either 454 or illumuna based platforms respectively and aligned to the human genome using the University of California, Santa Cruz BLAST Like Alignment Tool, BLAT (hg18, version 36.1). Enrichment of the sterol regulatory complex genes was calculated by comparing how often that gene was mutated in the screen to how often the gene carries an insertion in the control library data set. For each sterol regulatory complex gene a *P*-value was calculated using the one-sided Fisher exact test.

### siRNA screen

siRNAs from the Ambion Druggable genome library representing 9,102 genes were spotted in 54 384-well white bottom plates in a 2×2 format such that each gene was targeted by 2 different pools of 2 siRNAs - 4 unique siRNAs in total. Positive and negative control siRNAs were plated in triplicate on each plate. Using a liquid handler (WellMate, Thermo Fisher) to decrease variability, 0.5 µL of HiPerFect (Qiagen) in 9.5 µL of OptiMem (Gibco) was added to each well and incubated for 15 min at room temperature to allow complex formation. HEK293T/ffLuc cells per well were plated to achieve a 40 nM final siRNA concentration. 72 hours post-transfection, cells were infected with VSV-(ANDV)*rLuc. 24 hrs post-infection, firefly and Renilla luciferase expression were measured. Robust z-scores were calculated for each plate using the median and interquartile ranges of log-transformed RLUs [61]. For the secondary screen, 3 unique siRNAs for each gene, different from those used in the primary screen, were obtained from Ambion and arrayed in 96-well plates.

### Viral infection assays and inhibitor studies

Sub-confluent cells were spin-infected (45 minutes at 1200 × g, 20°C) with viral pseudotypes and harvested for infectivity assays 8–12 hr post-infection. Infected cells were quantified by FACS using RFP expression or staining with antibodies against the matrix protein of VSV [62], [63] followed by a secondary antibody conjugated to AF-647. At least 10^4^ events, in duplicate, were counted for at least three independent experiments. For PF-429242 and mevastatin studies, cells were pretreated for 24 hr before infection. All infections and overlays were carried out in the continued presence of drug or DMSO, for the length of the infection. For MβCD studies, cells were pretreated for one hour and washed prior to infection. Cells were infected with ANDV at an MOI of 3 by adding 1 mL of inoculum to a 6 well dish for two hours at 37°C. Viral inoculum was removed, cells were overlaid with fresh media containing drug (except for MβCD assays) or DMSO where indicated. Cells were harvested three to four days post-infection, fixed for one hour in 4% formaldehyde then analyzed by flow cytometry using anti-ANDV N.

### Virus binding and internalization

rVSV-ANDV binding was performed at 4°C in 24 well plates of HAP1 cells in IMDM containing a 1∶8 ratio of 10% FBS to delipidated FBS. After one hour on ice, cells were washed with ice-cold PBS to remove unbound virus, and samples to measure bound virus were collected by scraping cells into PBS, followed by additional washing. Trypsin-EDTA was used to remove surface bound virus. rVSV-ANDV internalization was measured by first binding and washing at 4°C then incubating samples at 37°C for one hour in IMDM containing delipidated media. Cells were then washed with PBS and treated with 0.05% trypsin and washed to remove surface bound virus. Samples were kept on ice after the final washing step, then processed for RNA. Primers specific to the VSV N segment were used for qRT-PCR. Data were analyzed using the ΔΔCT method [64] by calculating the change in gene expression normalized to that of GAPDH as a housekeeping gene.

### TALEN-mediated disruption of SCAP and ANDV-VSV infection

A TALEN pair targeting exon 3 in SCAP was designed and constructed as previously described [65]. Mutations induced by non-homologous end joining (NHEJ) following expression of the SCAP TALEN were measured as previously described [17]. Band intensities were quantified using ImageJ and utilized to estimate mutation rates as previously described using the formula: % gene modification = 100×(1-(1-fraction cleaved)^1/2^) [66]. The T7 endonuclease assay has a range of detection from approximately 1% to 50% NHEJ.

More detailed Methods descriptions are given in the Supplemental Information.

## Supporting Information

Figure S1 **Susceptibility of rVSV-ANDV-selected population.** Parental HAP1 and mutagenized rVSV-ANDV surviving HAP1 (rVSV-ANDV^R^) cells were infected with rVSV and rVSV-ANDV. Cells were harvested 12 hpi and infection was quantified by viral protein expression using flow cytometry analysis. Values presented are relative infection levels in rVSV-ANDV^R^ cells compared to parental HAP1 cells.
(PDF)

Figure S2 **Validation of siRNA screen controls.** Non-targeting negative control and positive controls targeting ffLuc, rLuc, and the endosomal proton pump member ATP6V06 were plated in triplicate on each plate. Cytotoxic siDeath was included as a control for both cell viability and infection. Values are shown as percent relative to negative control. Using a z-score<1.5 cutoff, >95% of controls were correctly identified in the primary screen.
(PDF)

Figure S3 **Validation of S1P, S2P, and SCAP depletion in CHO mutants.** Reverse Transcriptase PCR analysis of SREBF2, SCAP, S1P, and S2P transcripts from Chinese Hamster Ovary cell lines of wild type (CHO-K1), -MBTPS1 (S1P), -MBTPS2 (S2P), and SCAP –mutant cell lines. Cell lines were additionally validated via Western blot analysis (not shown). Low levels of SCAP transcripts in the CHO_SCAP-_ cells are likely due to a propensity towards reversion.
(PDF)

Figure S4 **SCAP TALEN induced mutation sequences.** SCAP-TALEN induced mutations were identified by PCR amplification of genomic DNA from SCAP-TALEN treated cells pre- and post-infection with ANDV. PCR amplicons were TOPO cloned (Invitrogen) and sequenced using the SP6 primer. 18% (4/22) of clones sequenced prior to infection had mutations at the TALEN cut-site, whereas 100% (24/24) of clones sequenced after infection showed evidence of TALEN-induced mutations. The SCAP-TALEN cut-site is shown in bold font. The column on the right represents number of bases inserted or deleted. Base insertions and substitutions are shown in red.
(PDF)

Figure S5 **Total cellular cholesterol in PF-429242-treated Vero E6 cells.** Measurement of cholesterol in Vero E6 cells pretreated with PF-429242 or vehicle (DMSO) for 24 hours shown relative to untreated cells. Mean±SEM shown for three independent experiments; p<0.03 relative to control sample with all drug doses greater than 1.25 µM.
(PDF)

Figure S6 **Kinetics of viral entry in cholesterol-depleted cells.** Vero E6 (A) and A549 (B) cells were pretreated with the S1P inhibitor PF-429242 (20 µM) for 24 hours prior to infection. Virus was bound at 4°C via spinoculation for 30 minutes, then warmed to 37°C to allow entry to occur. At 3.5 hpi, ammonium chloride (NH_4_Cl) was added to block any subsequent viral fusion. Cells were fixed at 10 hpi, immunostained for VSV M production, and infection quantified by flow cytometry.
(PDF)

Figure S7 **S1P expression analysis.** Lysates from wild-type HAP1 cells (HAP1_WT_), a HAP1 clone containing a gene-trap integration into *S1P* (HAP1_S1P_), and 293T cells overexpressing S1P by transient transfection (293T cmv-S1P) were subject to western blot analysis.
(PDF)

Figure S8 **Analysis of ANDV entry kinetics.** An ammonium chloride time-of-addition assay was used to analyze kinetics of ANDV glycoprotein mediated entry. (A) Wildtype HAP1 (HAP1_WT_) or (B) S1P null (HAP1_S1P_) cells were chilled on ice and infected with rVSV-ANDV at an MOI of 10 at 4 degrees to allow virus to bind. Cells were warmed quickly at 37 to initiate a synchronous infection and 50 mM NH_4_Cl was added at the indicated times post warming. Cells were fixed at 14 h.p.i. and stained for VSV-M to visualize infected cells (red, VSV-M; blue, nuclei).
(PDF)

Figure S9 **Control for DiO labeled virion preparation.** Supernatant from mock-infected 293T cells was collected, purified, concentrated, and labeled with DiO lipophilic dye (DiO-488, as described in the Supplemental Information) to label any lipid-containing debris or microsomes. Labeled stocks where then allowed to *bind* to Vero E6 cells at 4°C, incubated at the indicated times at 37°C, fixed with paraformaldehyde, stained with Wheat Germ Agglutinin (WGA-647), mounted and deconvolution microscopy performed. Representative fields are provided.
(PDF)

Methods S1 **Expanded materials and methods.** Plasmids, viruses, and cell lines used; HAP1 library generation, selection, and integration site mapping; siRNA screen, qRT-PCR, viral binding and internalization, cholesterol analysis, T7 endonuclease assay, and viral particle labeling and microscopy.
(DOCX)

Table S1 **Gene-trap integration site analysis in HAP1 cells selected with rVSV-ANDV.** Gene symbol, chromosomal position and orientation, and number of independent integration events into each, are listed. Intragenic integrations are ranked based on observed frequency.
(XLSX)

Table S2 **Candidate genes identified in siRNA primary screen as required for VSV-(ANDV) infectivity.** Gene symbol, ID, Accession Number, and full gene name are tabulated. Z-scores of two pairs (AB and CD) of siRNAs, each for infection (rLuc) and cell viability (ffLuc) have been determined (as per materials and methods). Those genes that were validated are listed as such (‘yes’ or ‘no’).
(XLSX)

Table S3 **Relative Infectivity of cell lines used.** Numbers indicate the fold difference in viral input, relative to Vero E6 cells, required to achieve ∼30% infectivity in the indicated cells lines.
(PDF)
