# Supplementary figures and images for: The Major Cellular Sterol Regulatory Pathway Is Required for Andes Virus Infection
Source: PLoS Pathog. 2014 Feb 6;10(2):e1003911. doi: 10.1371/journal.ppat.1003911 (PMC3916400; doi:10.1371/journal.ppat.1003911)

Figure S1: Susceptibility of rVSV-ANDV-selected population

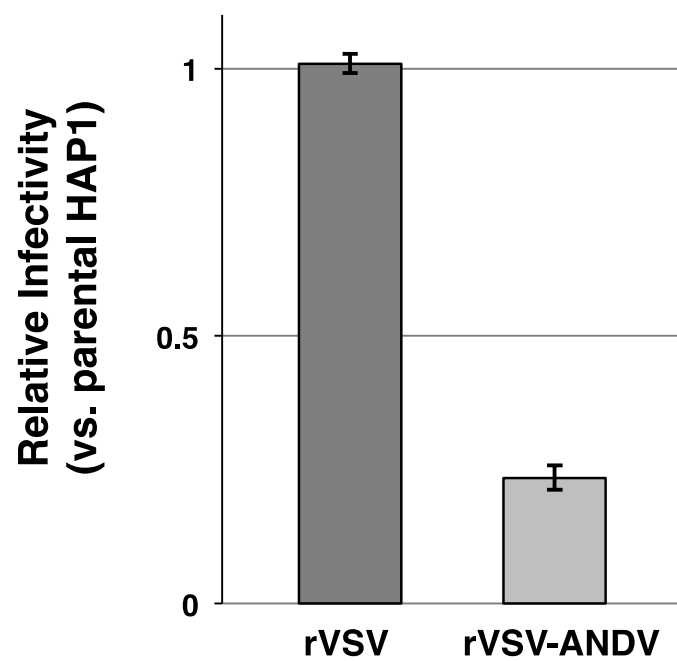

Supplement: Figure S1 — Susceptibility of rVSV-ANDV-selected population. Parental HAP1 and mutagenized rVSV-ANDV surviving HAP1 (rVSV-ANDVR) cells were infected with rVSV and rVSV-ANDV. Cells were harvested 12 hpi and infection was quantified by viral protein expression using flow cytometry analysis. Values presented are relative infection levels in rVSV-ANDVR cells compared to parental HAP1 cells. (PDF) [file ppat.1003911.s001.pdf]

Figure S2. Validation of siRNA Screen Controls

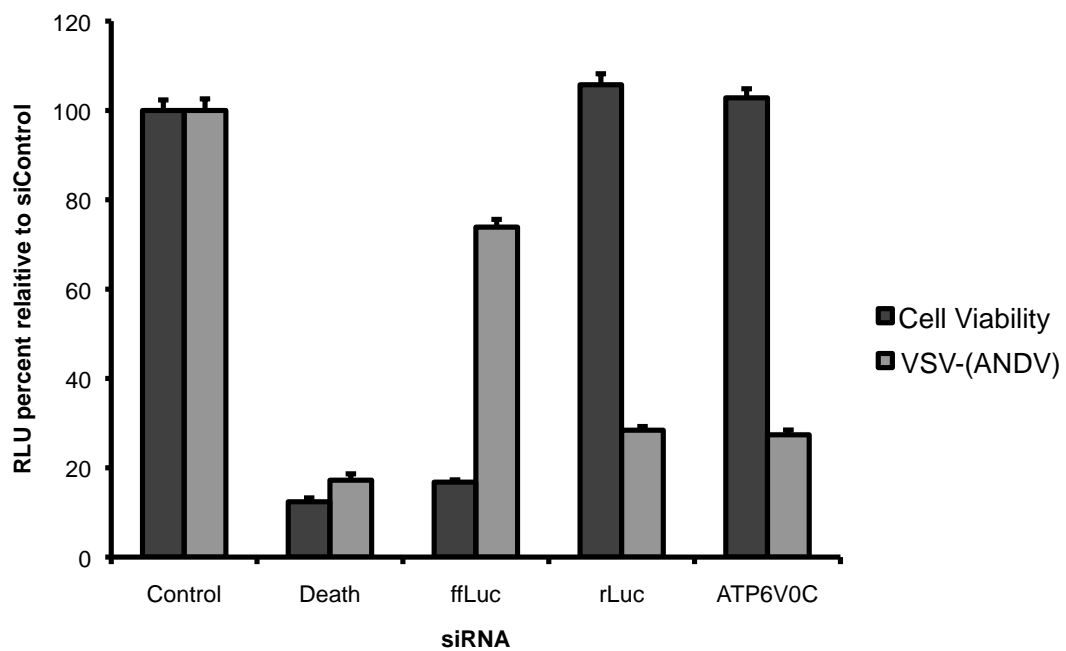

Supplement: Figure S2 — Validation of siRNA screen controls. Non-targeting negative control and positive controls targeting ffLuc, rLuc, and the endosomal proton pump member ATP6V06 were plated in triplicate on each plate. Cytotoxic siDeath was included as a control for both cell viability and infection. Values are shown as percent relative to negative control. Using a z-score<1.5 cutoff, >95% of controls were correctly identified in the primary screen. (PDF) [file ppat.1003911.s002.pdf]

Figure S3. Validation of S1P, S2P, and SCAP depletion in CHO mutants

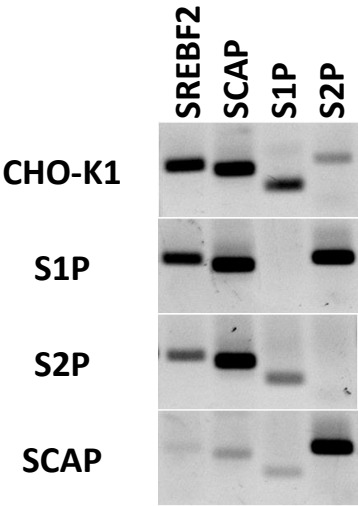

Supplement: Figure S3 — Validation of S1P, S2P, and SCAP depletion in CHO mutants. Reverse Transcriptase PCR analysis of SREBF2, SCAP, S1P, and S2P transcripts from Chinese Hamster Ovary cell lines of wild type (CHO-K1), -MBTPS1 (S1P), -MBTPS2 (S2P), and SCAP –mutant cell lines. Cell lines were additionally validated via Western blot analysis (not shown). Low levels of SCAP transcripts in the CHOSCAP- cells are likely due to a propensity towards reversion. (PDF) [file ppat.1003911.s003.pdf]

Figure S5. Total cellular cholesterol in PF-429242-treated Vero E6 cells

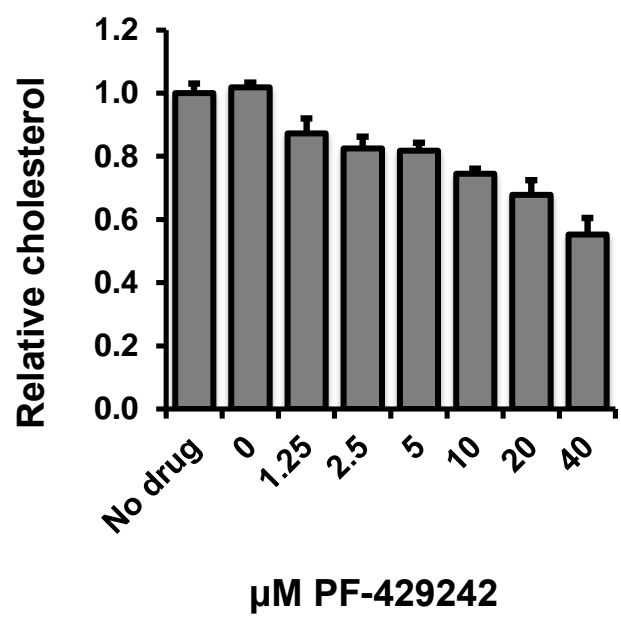

Supplement: Figure S5 — Total cellular cholesterol in PF-429242-treated Vero E6 cells. Measurement of cholesterol in Vero E6 cells pretreated with PF-429242 or vehicle (DMSO) for 24 hours shown relative to untreated cells. Mean±SEM shown for three independent experiments; p<0.03 relative to control sample with all drug doses greater than 1.25 µM. (PDF) [file ppat.1003911.s005.pdf]

Figure S6. Kinetics of viral entry in cholesterol-depleted cells

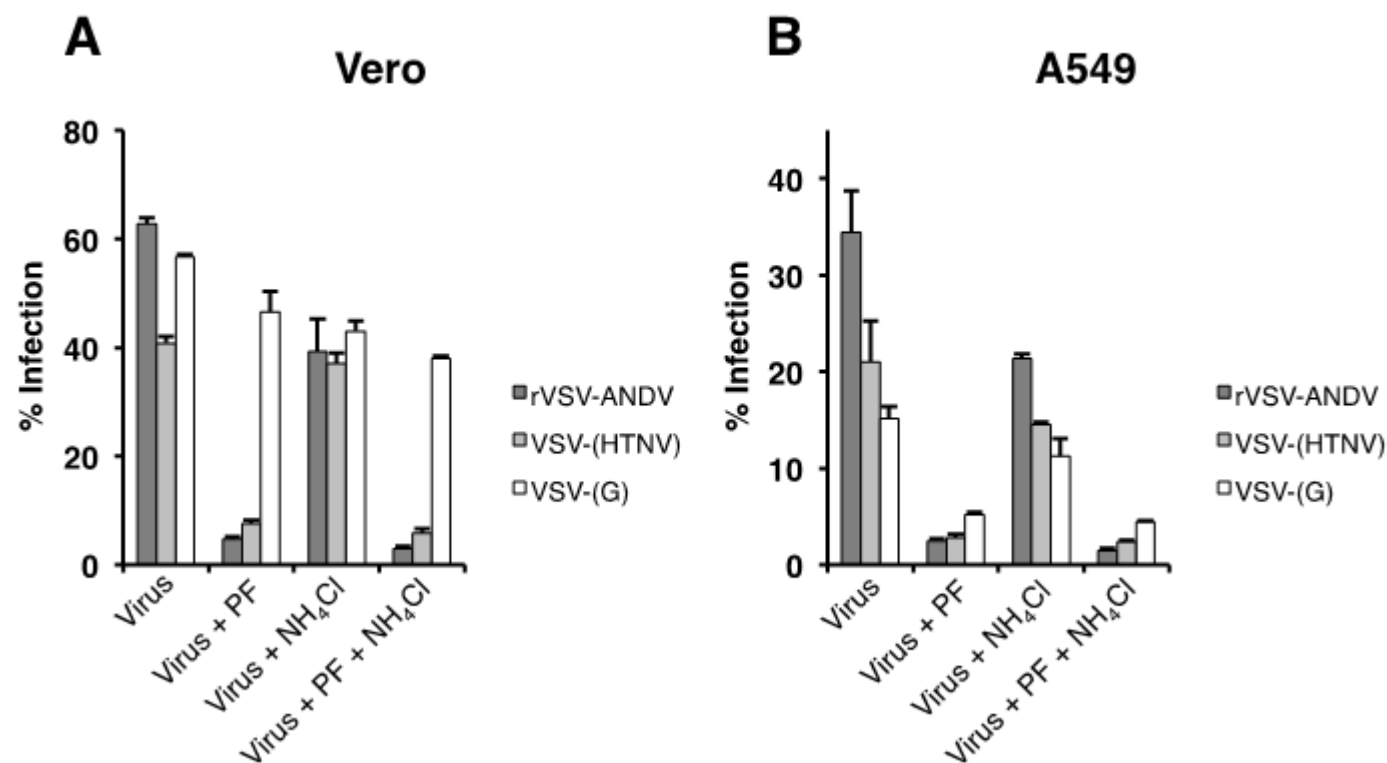

Supplement: Figure S6 — Kinetics of viral entry in cholesterol-depleted cells. Vero E6 (A) and A549 (B) cells were pretreated with the S1P inhibitor PF-429242 (20 µM) for 24 hours prior to infection. Virus was bound at 4°C via spinoculation for 30 minutes, then warmed to 37°C to allow entry to occur. At 3.5 hpi, ammonium chloride (NH4Cl) was added to block any subsequent viral fusion. Cells were fixed at 10 hpi, immunostained for VSV M production, and infection quantified by flow cytometry. (PDF) [file ppat.1003911.s006.pdf]

Figure S7. S1P expression analysis

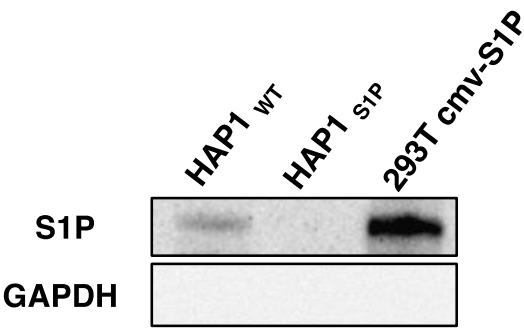

Supplement: Figure S7 — S1P expression analysis. Lysates from wild-type HAP1 cells (HAP1WT), a HAP1 clone containing a gene-trap integration into S1P (HAP1S1P), and 293T cells overexpressing S1P by transient transfection (293T cmv-S1P) were subject to western blot analysis. (PDF) [file ppat.1003911.s007.pdf]

Figure S8. Analysis of ANDV entry kinetics.

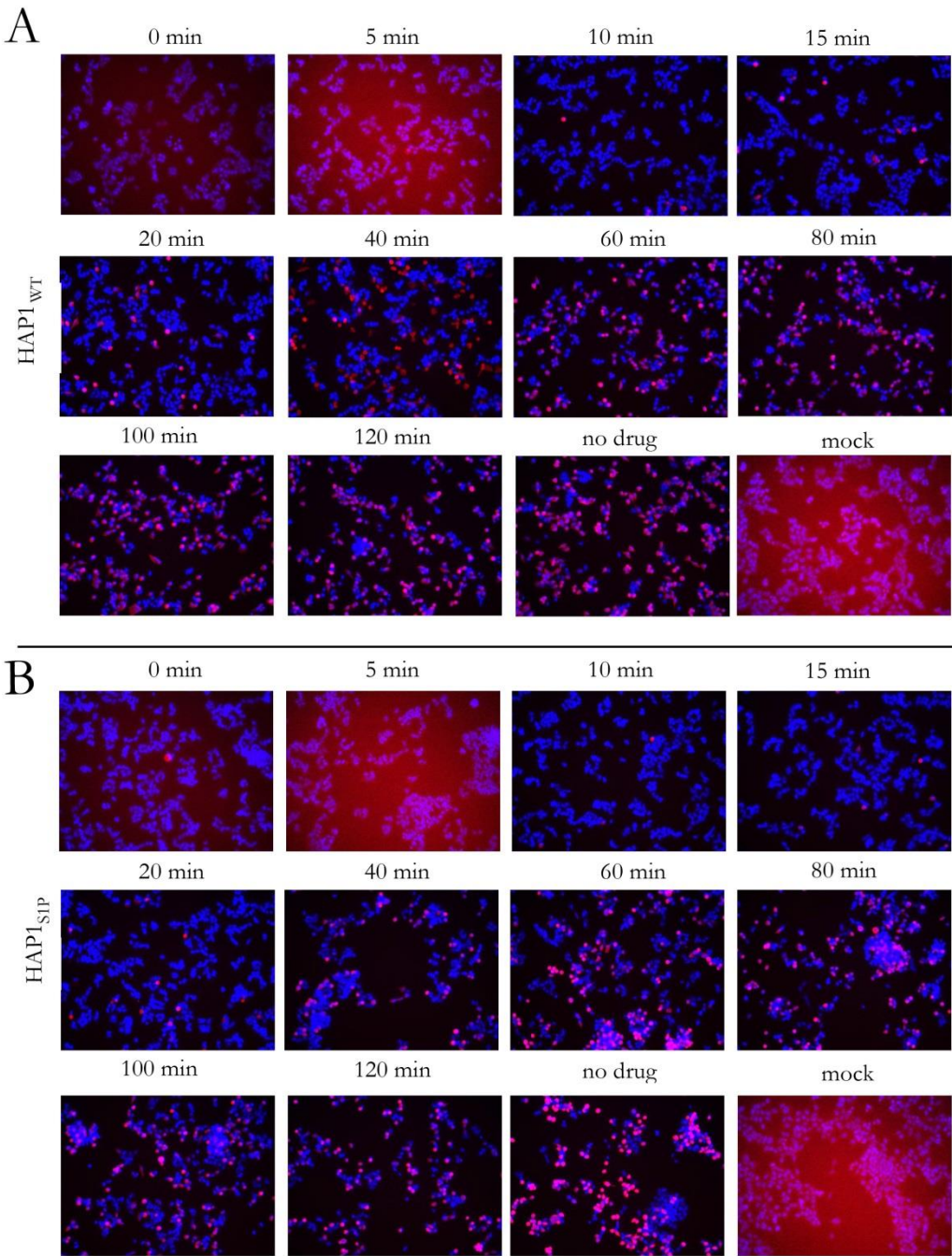

Supplement: Figure S8 — Analysis of ANDV entry kinetics. An ammonium chloride time-of-addition assay was used to analyze kinetics of ANDV glycoprotein mediated entry. (A) Wildtype HAP1 (HAP1WT) or (B) S1P null (HAP1S1P) cells were chilled on ice and infected with rVSV-ANDV at an MOI of 10 at 4 degrees to allow virus to bind. Cells were warmed quickly at 37 to initiate a synchronous infection and 50 mM NH4Cl was added at the indicated times post warming. Cells were fixed at 14 h.p.i. and stained for VSV-M to visualize infected cells (red, VSV-M; blue, nuclei). (PDF) [file ppat.1003911.s008.pdf]

Figure S9. Control for DiO labeled virion preparation.

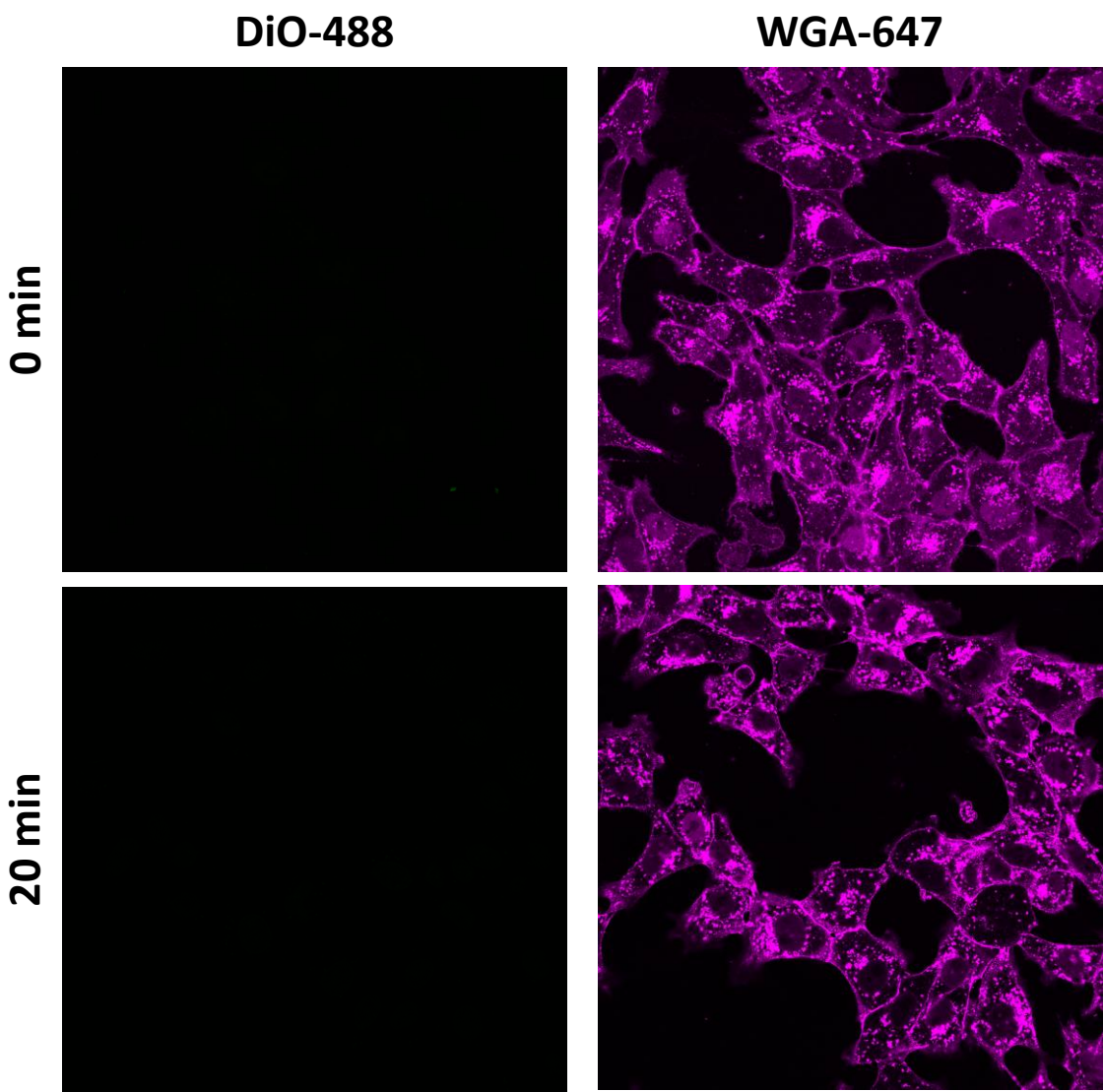

Supplement: Figure S9 — Control for DiO labeled virion preparation. Supernatant from mock-infected 293T cells was collected, purified, concentrated, and labeled with DiO lipophilic dye (DiO-488, as described in the Supplemental Information) to label any lipid-containing debris or microsomes. Labeled stocks where then allowed to bind to Vero E6 cells at 4°C, incubated at the indicated times at 37°C, fixed with paraformaldehyde, stained with Wheat Germ Agglutinin (WGA-647), mounted and deconvolution microscopy performed. Representative fields are provided. (PDF) [file ppat.1003911.s009.pdf]
