# Supplementary material for: The Major Cellular Sterol Regulatory Pathway Is Required for Andes Virus Infection
Source: PLoS Pathog. 2014 Feb 6;10(2):e1003911. doi: 10.1371/journal.ppat.1003911 (PMC3916400; doi:10.1371/journal.ppat.1003911)
Supplement: Figure S4 — SCAP TALEN induced mutation sequences. SCAP-TALEN induced mutations were identified by PCR amplification of genomic DNA from SCAP-TALEN treated cells pre- and post-infection with ANDV. PCR amplicons were TOPO cloned (Invitrogen) and sequenced using the SP6 primer. 18% (4/22) of clones sequenced prior to infection had mutations at the TALEN cut-site, whereas 100% (24/24) of clones sequenced after infection showed evidence of TALEN-induced mutations. The SCAP-TALEN cut-site is shown in bold font. The column on the right represents number of bases inserted or deleted. Base insertions and substitutions are shown in red. (PDF) [file ppat.1003911.s004.pdf]

Figure S4. SCAP TALEN induced mutation sequences

SCAP, Pre-infection 18% mutated (4/22 clones)

AGTCATTCTGCCAGAAGT**TCCCAGGGGACAGCAGC**AGGCATCCATGCTCAGGGAGTAGGTT	w.t.
AGTCATTCTGCCAGAAGTTCCCAGGG-ACAGCAGCAGGCATCCATGCTCAGGGAGTAGGTT	-1
AGTCATTCTGCCAGAAGTTCCCAGGGGA**GGA**CAGCAGCAGGCATCCATGCTCAGGGAGTAG	+3
AGTCATTCTGCCAGAAGTTCCCAGGGG**G**ACAGCAGCAGGCATCCATGCTCAGGGAGTAGGT	+1

SCAP, Post-infection 100% mutated (24/24 clones)

AGTCATTCTGCCAGAAGT**TCCCAGGGGACAGCAGC**AGGCATCCATGCTCAGGGAGTAGGTT	w.t.
AGTCATTCTGCCAGAAGTTCCCAGGG-ACAGCAGCAGGCATCCATGCTCAGGGAGTAGGTT	-1
AGTCATTCTGCCAGAAGTTCCCAGG**A**GA--GCAGCAGGCATCCATGCTCAGGGAGTAGGTT	-2
AGTCATTCTGCCAGAAGTTCC----GGACAGCAGCAGGCATCCATGCTCAGGGAGTAGGTT	-4
AGTCATTCTGCCAGAAGTTCCCAG-------CAGCAGGCATCCATGCTCAGGGAGTAGGTT	-7
AGTCATTCTGCCAGAAGTTC--------CAGCAGCAGGCATCCATGCTCAGGGAGTAGGTT	-8
AGTCATTCTGCCAGAAGTTCC--------A**A**CA**C**C**TC**GC**TG**CC**C**TGCTCAGGGAGTA**C**GGT	-8
AGTCATTCTGCCAGAAGTTCCCAG----------CAGGCATCCATGCTCAGGGAGTAGGTT	-10
AGTCATTCTGCCAGAAGTTCCCAGG-------------CATCCATGCTCAGGGAGTAGGTT	-13
AGTCATTCTGCCAGAAGTTCCCAG--------------CATCCATGCTCAGGGAGTAGGTT	-14
