# Supplementary material for: The Major Cellular Sterol Regulatory Pathway Is Required for Andes Virus Infection
Source: PLoS Pathog. 2014 Feb 6;10(2):e1003911. doi: 10.1371/journal.ppat.1003911 (PMC3916400; doi:10.1371/journal.ppat.1003911)
Supplement: Methods S1 — Expanded materials and methods. Plasmids, viruses, and cell lines used; HAP1 library generation, selection, and integration site mapping; siRNA screen, qRT-PCR, viral binding and internalization, cholesterol analysis, T7 endonuclease assay, and viral particle labeling and microscopy. (DOCX) [file ppat.1003911.s010.docx]

**Supplemental Methods**

**Plasmids**

For gene-trap mutagenesis, a new HIV-based genome plasmid, pLentiET, was constructed. pLentiET is a derivative of the MLV-based plasmid, pRET.IL.IRES-eGFP (no HSV-TK) ([Ishida and Leder 1999](#_ENREF_21)). pLentiET was constructed as follows: pRET.IL.IRES-eGFP was digested with Sal I, liberating a 4 kb fragment containing sequences essential for gene splicing, *egfp* and *neo* expression. This fragment was subsequently blunted with T4 DNA polymerase (NEB) and TOPO cloned (TOPO-Blunt II, Invitrogen). Following PCR amplification using primers with unique restriction sites and restriction digest with Xma I and Cla I, this 4 kb fragment was ligated into pWPT-GFP (Addgene) previously digested with Xma I and Cla I generating the final pLentiET construct.

Pseudovirion plasmids encoding the full length VSV Indiana genome where the VSV glycoprotein (G) has been replaced with either renilla luciferase, firefly luciferase, or RFP have been previously described ([Ray, Whidby et al. 2010](#_ENREF_42)). The pCAGGS vector expressing codon-optimized HNTV was synthesized (GeneArt). pCAGGS-SINV was a kind gift of Graham Simmons (University of California San Francisco, CA).

|  |  |  |  |
| --- | --- | --- | --- |
| **Oligo Name** | **Function** | **Target** | **Sequence (5' -> 3')** |
| Exon-trap forward (XmaI) | pLentiET generation |  | AAATTCCCGGGCTTTATGCTTCCGGCTCGTATGTTGTGTGG |
| Exon-trap reverse (ClaI) | pLentiET generation |  | AATTTATCGATGGGATGTGCTGCAAGGCGATTAAGTTGGG |
| A-tail Linker | deep sequencing |  | TATGCGGGACAGGTAATACGCGAGCCAGACTCCATATTCTCCGCTTAAGGGACT |
| HIV LTR 1 | deep sequencing | HIV LTR | CTTAAGCCTCAATAAAGCTTGCCTTGAG |
| HIV LTR 2 Barcode | deep sequencing | HIV LTR | gccttgccagcccgctcagXXXXXXXXAGACCCTTTTAGTCAGTGTGGAAAATC |
| Linker-PCR 1 | deep sequencing | Linker | TATGCGGGACAGGTAATACGCG |
| Linker-PCR 2 | deep sequencing | Linker | gcctccctcgcgccatcagACGCGAGCCAGACTCCATATT |
| ffLuc siRNA | siRNA | ffLuc | GGACGAGGACGAGCACUUCUU |
| rLuc siRNA 1 | siRNA | rLuc | GAUCAAAUCUGAAGAAGGAUU |
| rLuc siRNA2 | siRNA | rLuc | CGUGAAAUCCCGUUAGUAAUU |
| SREBF2 si-1 | siRNA | SREBF2 | GCGCTCTCATTTTACCAAA |
| SREBF2 si-2 | siRNA | SREBF2 | CCGCAGTGTCCTGTCATTC |
| VSV M Forward | qRT-PCR | VSV M | CGGAGGATTGACGACTAATGC |
| VSV M Reverse | qRT-PCR | VSV M | ACCATCCGAGCCATTCGA |
| VSV M Probe (FAM/MGB) | qRT-PCR | VSV M | CGCCACAAGGCAG |
| SCAP Forward | TALEN - T7 Endonuclease Assay | SCAP | CTTGTCCTGTCTCCTGTGCAG |
| SCAP Reverse | TALEN - T7 Endonuclease Assay | SCAP | CACACATCAAAAAGACGGTGAC |

**Cell lines**

Unless otherwise stated, all cell lines were kept at 37°C in 5% CO_2_. HAP1 cells, a derivative of the human myeloid leukemia cell line, KBM7 ([Carette, Raaben et al. 2011](#_ENREF_7)), were maintained in Iscove's Modified Dulbecco's Medium (IMDM) supplemented with 10% fetal bovine serum (FBS), and standard concentrations of sodium pyruvate, penicillin, streptomycin, and sodium glutamate (Invitrogen). CHO cell lines null for S1P, SCAP (a kind gift of Michael Brown and Joseph Goldstein, University Texas Southwestern, ([Hua, Nohturfft et al. 1996](#_ENREF_20), [Rawson, Cheng et al. 1998](#_ENREF_41))), and S2P (a kind gift of TY Chang, Dartmouth Medical School, ([Sakai, Duncan et al. 1996](#_ENREF_48))) were maintained in a 1:1 mixture of Ham's F12:DMEM supplemented with 10% FBS, penicillin, streptomycin, 5 μg/ml cholesterol (Sigma), 50 μM sodium mevalonate, and 20 μM sodium oleate. CHO cells were regularly selected with 50 μg/ml Amphotericin B (Sigma) as previously described ([Sakai, Duncan et al. 1996](#_ENREF_48)). Human A549 cells were maintained under standard conditions in DMEM supplemented with 10% FBS, and standard concentrations of sodium pyruvate, penicillin, streptomycin, and sodium glutamate (Invitrogen).

**Haploid cell enrichment and generation of HAP1 library**

HAP1 cells were routinely assessed for ploidy indirectly by Hoechst 33342 staining of Triton X-100 –released nuclei on a flow cytometer. To separate haploid HAP1 cells from those that had reverted to diploid, we took advantage of cell size differences between these two populations. Haploid HAP1 cells grew to a smaller average diameter than diploid HAP1 cells. These two populations were separated either by automated cell sorting based solely on light scatted and/or using cell sieving by pouring mixed populations through a molecular mesh (BioDesign Inc.) with a 5 µM porosity. The latter resulted in population enrichment to near 80% haploid without the cell death associated with cell sorting.

A large insertionally-mutagenized library of HAP1 cells (~1 x 10^9^ cells, ~80% haploid) was produced as follows. HAP1 cells were plated into 6-well tissue culture plates coated with collagen IV to achieve a density roughly 60% at the time on infection. Gene-trap mutagenesis was carried out in 3 successive rounds of spin infections (60 minutes at 1200 x g, 12 hour intervals between each infection) using enough pLentiET gene-trap virus to achieve an infectivity of approximately 80% as judged by parallel experiments with a GFP reporter virus, normalized to pLentiET viral stocks by P24 protein levels. This HAP1 library was later expanded for at least 6 days and aliquots frozen in DMSO for future screens.

**HAP1 library selection**

Frozen stocks of HAP1 cells (~75 x 10^6^ cells) were plated onto collagen IV coated tissue culture plates selected with either rVSV-VSV (MOI of 2) or rVSV-ANDV (MOI of 3-5). Cells selected for resistance to rVSV-ANDV were collected and pooled within 3 weeks and saved as either DMSO frozen stocks or for chromosomal DNA preparation. Clonal populations of HAP1 cells were achieved by limiting dilution.

**Integration site mapping**

DNA amplicon preparation was carried out as previously described ([Wang, Ciuffi et al. 2007](#_ENREF_51)). Briefly, chromosomal DNA was prepared from either pools or clonal populations of ANDV resistant HAP1 cells using the DNeasy Blood and Tissue DNA Extraction kit (Qiagen). Chromosomal DNA preparations were next fragmented with a commercial blend of DNA endonucleases (Fragmentase, NEB), as per manufacturer’s protocols, to achieve an average size distribution of 500 bp. Fragmented DNA was purified using a Qiaquick PCR Purification kit (Qiagen), blunted with T4 polymerase (NEB), and A-tailed with Klenow Fragment (NEB). Previously-annealed linkers (see primer table below) were ligated to amplicon pools using Quick Ligase (NEB), and used as a PCR template. The product of this initial PCR was subsequently diluted and used as template in a second, nested PCR. This second PCR utilized primers containing unique 6 bp barcode regions for sample identification and a sequencing primer annealing site. Amplicons generated from this last PCR were agarose gel purified (Qiaquick Gel Extraction kit, Qiagen) and subjected to AMPure Bead purification (Beckman Coulter), as per manufacturer's instructions. For clonal HAP1 populations, amplicons were cloned into TOPO-Blunt II (Invitrogen) and plasmid DNA sent for standard DNA sequencing analysis. Amplicons derived from complex pools of ANDV resistant cells were subjected to 454 sequencing analysis using a GS Junior sequencer (Roche). 454 reads were first decoded by requiring a perfect match to the sample barcode and subsequently trimmed. The resulting collection of reads were quality filtered by requiring a 95% match to the LTR primer, and 100% match to the flanking LTR region. Only sequences that began within three base pairs of the LTR end and showed unique best alignments to the human genome by the University of California, Santa Cruz BLAST Like Alignment Tool, BLAT (hg18, version 36.1, >98% match score) were considered true integration sites.

**siRNA screen**

HEK293T cell line expressing firefly luciferase (HEK293T /ffLuc) was created by transfecting HEK293T /17 cells with pGL4.27 plasmid (Promega) and selecting with Hygromycin B at 200µg/mL. Clones were chosen based on highest levels of firefly luciferase expression. AllStars Negative Control siRNA (Qiagen) and AllStars Hs Cell Death siRNA were obtained from Qiagen. siRNA targeting Firefly Luciferase from pGL4.27 vector (GGACGAGGACGAGCACUUCuu) and siRNA targeting Renilla Luciferase was a pool of 2 sequences (GAUCAAAUCUGAAGAAGGAuu, CGUGAAAUCCCGUUAGUAAuu). ON-TARGETplus pooled siRNA targeting ATP6V0C was obtained from Dharmacon. siRNAs from the Ambion Druggable genome library representing 9,102 genes were spotted in 54 384-well white bottom plates. siRNAs were arrayed in a 2x2 format such that each gene was targeted by 2 different pools of 2 siRNAs - 4 unique siRNAs in total. Each of the positive and negative control siRNAs were plated in triplicate on each plate. Using a liquid handler (WellMate, Thermo Fisher) to decrease variability, 0.5µL of HiPerFect (Qiagen) in 9.5µL of OptiMem (Gibco) was added to each well and incubated for 15min at room temperature to allow complex formation. HEK293T /ffLuc cells per well were plated to achieve a 40nM final siRNA concentration. 72 hours post-transfection, cells were infected with VSV-(ANDV)*rLuc. 24hrs post-infection, firefly and renilla luciferase expression were measured using Brite-Glo and the Stop&Glo Reagent component of the Dual Glo Luciferase System. Relative Light Units were measured. Robust z-scores were calculated for each plate using the median and interquartile ranges of log-transformed RLUs. For the secondary screen, the same procedure was used as in the primary screen, but volumes were scaled up 2.5-fold for 96-well plates. 3 unique siRNAs for each gene, different from those used in the primary screen, were obtained from Ambion and arrayed in 96-well plates. Positive and negative controls were included on each plate.

**SREBF2 knockdown**

siRNAs targeting SREBF2, si-1 (Ambion, s27) and si-2 (Qiagen, SI00065856), were plated in a 12-well format and complexed with HiPerFect in OptiMem. HEK293T cells were reverse-transfected onto siRNA complexes for a 40nM final siRNA concentration. Cells were incubated for 72hrs to allow for knockdown. Cells were then infected with VSV-(ANDV)*RFP, VSV-(HTNV)*RFP, or VSV-(VSV)*RFP pseudovirions. 18hrs post-infection, cells were fixed in 1% paraformaldehyde and RFP expression was measured by flow cytometry.

**Viral infection assays and inhibitor studies**

For all infection assays, sub-confluent cells were infected with the indicated viruses. Virus-containing media was spin-infected (45 minutes at 1200 x g, 20°C) and cells harvested for infectivity assays 8-12 hrs post-infection. Infected cells were quantified for the expression of RFP or stained with antibody directed against the matrix of VSV-M ([Lefrancois and Lyles 1982](#_ENREF_25), [Lefrancois and Lyles 1982](#_ENREF_26)) for rVSV-ANDV or Large T antigen (Calbiochem) of SV-40, stained with a secondary antibody conjugated to AF-647, and then analyzed by flow cytometry using an LSR Flow Cytometer (BD Biosciences). At least 10^4^ events, in duplicate, were counted for at least three independent experiments. For PF-429242 and mevastatin studies, cells were pretreated with the indicated concentrations of PF-429242 (Tocris Biosciences) or mevastatin (Sigma-Aldrich) in DMSO for 24 hr before infection. All infections and overlays were carried out in the continued presence of drug or DMSO, for the length of the infection. Cells were pretreated with MβCD (Sigma-Aldrich) for one hour and washed prior to infection. Cells were infected with Andes virus at an MOI of 3 for two hours at 37°C. Viral inoculum was removed, cells were overlaid with fresh media containing drug (except for MβCD assays) or DMSO where indicated. Cells were harvested for flow cytometry three to four days post-infection and fixed for one hour in 4% formaldehyde. Cells were permeabilized and stained with rat anti-ANDV N sera (1:300, courtesy of Dr. Andrew Pekosz, ([Rowe, Suszko et al. 2008](#_ENREF_47))) and a fluorescently-conjugated secondary antibody (1:1000, anti-Rat 647 Invitrogen A-21247). Infected cells were quantified using a LSR flow cytometer (BD Biosciences) and 10^5^ events were collected in triplicate.

**Cell lysis & Western Blot analysis**

Cells were collected for western blot analyses at the times indicated. Cell lysates were prepared by whole-cell solubilization in either RIPA buffer (10 mM Tris-HCl pH 8.0, 5 mM EDTA, 140 mM NaCl, 1.0% w/v Sodium Deoxycholate, 0.1% SDS, 1.0% NP40) or 1% Triton-X 100 lysis buffer (50mM Tris, 150mM NaCl, 5mM EDTA) in the presence of a protease inhibitor cocktail (Roche). Samples were boiled in a denaturing buffer (250 mM Tris-HCl pH 6.8, 10% SDS, 1.0% v/v β-mercaptoethanol, 10% glycerol, and bromophenol blue) for 5 min and electrophoresed through 4-15% Criterion precast polyacrylamide gels (Bio-Rad). Proteins were blotted onto 0.45 μM Nitrocellulose membranes (Bio-Rad) using a wet-transfer system under standard conditions. Membranes were probed with either anti-SREBP-2 (Millipore) or anti-GAPDH (Calbiochem) at 1:5000 and 1:10000, respectively. Detections were accomplished using secondary antibodies conjugated to HRP that was cleaved using Femto detection reagent (Thermo Scientific).

**Virus Binding and Internalization**

HAP1 cells were seeded in 24 well plates in IMDM containing 10% FBS. 16 hours later media was replaced with IMDM containing a 1:8 ratio of 10% FBS to delipidated FBS (created through Cab-O-Sil treatment, Cabot Corporation, ([Weinstein 1979](#_ENREF_52))). 24 hours later the binding and internalization assay was performed. Cells were pre-chilled on ice at 4°C while rocking, rinsed with ice-cold PBS and incubated with rVSV-ANDV for one hour on ice. Samples were washed with ice-cold PBS to remove unbound virus, samples to measure bound virus were collected by scraping cells into PBS, followed by additional washing. Samples to measure background were washed with PBS and treated with 0.05% Trypsin-EDTA (Gibco) for ten minutes at 37°C. Cells were added to media containing FCS to quench trypsin activity and washed with PBS. Samples to measure internalized virus were washed with PBS and incubated in warm IMDM containing delipidated media (as referenced above) at 37°C for one hour to allow endocytosis. After the one-hour warming period, cells were washed with PBS and treated with 0.05% trypsin for ten minutes at 37°C. Cells were then added to media containing FCS and washed with PBS. Samples were kept on ice after the final washing step, and lysed according to the QIAGEN RNeasy Mini kit.

**RNA isolation and qRT-PCR**

Total RNA from cells was isolated using the QIAGEN RNeasy Mini kit according to the manufacturer's protocol. RNA was quantified by measuring absorbance at 260 nm. First-strand cDNA was generated from 1 μg of total RNA using the SuperScript VILO cDNA Synthesis Kit (Invitrogen) according to manufacturer's protocol. Primers specific to the VSV N segment (see primer table) were used for RT-PCR. Each reaction was then performed using an ABI 7500 real-time PCR system (Applied Biosystems) with the following conditions: (i) denaturation at 95°C for 20 sec; and (ii) 40 cycles of PCR amplification, with denaturation at 95°C for 3 sec and annealing and extension at 60°C for 30 sec. Data were analyzed using the ΔΔCT method ([Livak 2001](#_ENREF_27)) by calculating the change in gene expression normalized to that of GAPDH as a housekeeping gene.

**Cholesterol Quantification**

Cells were pretreated with PF-429242, mevastatin, MβCD or vehicle for the indicated times, harvested and washed 3-times in PBS. Lipid extraction was performed using chloroform:isopropanol:NP-40 (7:11:0.1). After centrifugation, the lipid-containing fraction was dried at 50°C and placed in a SpeedVac for an additional 30 minutes to evaporate trace amounts of organic solvent. Cholesterol content was measured using the Amplex Red Cholesterol Assay Kit (Invitrogen) according to the manufacturer’s protocol. Briefly, lipid fractions were resuspended in 1X reaction buffer and incubated with the provided enzymes and substrate for 30 minutes at 37°C. Fluorescence at 590nm was detected using FluoSTAR Optima 96-well plate reader (BMG Labtech). An unpaired t-test was used asses for statistical significance (p-value <0.03).

**TALEN-mediated disruption of SCAP and ANDV-VSV infection**

A TALEN pair targeting SCAP was designed to exon 3 and constructed with obligate heterodimer Fok1 catalytic domains (DDD and RRR) to minimize off-target disruption as previously described ([Dahlem, Hoshijima et al. 2012](#_ENREF_8)). The DNA binding sequence of SCAP TALEN-Left was TACTCCCTGAGCATGGATGCCT and that for SCAP TALEN-Right was TCCCAGTCATTCTGCCAGAAGT. The repeat variable disresidues were NI HD NG HD HD HD NG NN NI NN HD NI NG NN NN NI NG NN HD HD NG and HD HD HD NI NN NG HD NI NG NG HD NG NN HD HD NI NN NI NI NN NG, respectively.

**Quantification of TALEN-induced mutations using the T7 Endonuclease I assay**

Mutations induced by non-homologous end joining (NHEJ) following expression of the SCAP TALEN were measured as previously described

(Reyon et al., 2012). Briefly, genomic DNA was extracted from cells using the QIAamp DNA Blood mini kit (QIAGEN) and PCR-amplified using SCAP-specific primers (supplementary figure X) for 35 cycles. 16μl (~200ng) of the PCR product was mixed with NEBuffer 2 (New England Biolabs), denatured and reannealed in a thermocycler to generate wild-type and mutant DNA heteroduplexes using the following protocol: 95^o^C, 5min; 95-85 ^o^C at -2 ^o^C/s; 85-25 ^o^C at -0.1 ^o^C/s; hold at 4 ^o^C. PCR heteroduplex products were treated with 20U of T7 Endonuclease I (New England Biolabs) in a 20μl reaction and incubated at 37 ^o^C for 15 minutes. The reaction was stopped by the addition of 2μl 0.5M EDTA and reaction products were purified using the QIAquick PCR purification kit (QIAGEN) and run on a 2% agarose gel. Band intensities were quantified using ImageJ and used to estimate mutation rates as previously described (Guschin et al., 2010) using the formula: % gene modification = 100 x (1-(1-fraction cleaved)^1/2^). The T7 endonuclase assay has a range of detection from approximately 1% to 50% NHEJ.

**Viral particle labeling and confocal microscopy**

Virus-containing supernatants were collected from producing cells (Vero E6 or HEK 293T), clarified through centrifugation (5 minutes at 2000 RPM), filtered through a 0.45 µM filter, centrifuged through discontinuous sucrose gradient (20%/60%, ~20x concentration), and frozen at -80°C for later use. Samples were later stained with DiO (50nM final, Molecular Probes) for ~1hr at 4°C with shaking and used immediately for binding/internalization assays.

A549 cells that were previously plated onto #1.5 poly-d-lysine-coated coverslips were chilled to 4°C and DiO-labeled virus was added along with fresh DMEM (50µl virus: 150µl DMEM) to each well and binding allowed to proceed for 1hr. Following vigorous washing with chilled 1x PBS, cells were fixed for 30 minutes with 4% paraformaldehyde following the indicated incubations at 37°C (0 or 20 minutes). Following fixation, cells were stained for 30 minutes with Wheat Germ Agglutinan-647 (Molecular Probes), washed again with 1x PBS and mounted onto standard microscopy slides. Image acquisition was accomplished using a Leica TCS SP8 confocal inverted microscope run by LAS software (CDB University of Pennsylvania microscopy core). Image analysis was conducted using ImageJ. Fluorescence gain/image contrast was adjusted only to the extent that background/mock-infected/microsome controls remained dark. Displayed images are typical and representative of all those observed (~30 fields for each condition).
