# Supplementary material for: The Major Cellular Sterol Regulatory Pathway Is Required for Andes Virus Infection
Source: PLoS Pathog. 2014 Feb 6;10(2):e1003911. doi: 10.1371/journal.ppat.1003911 (PMC3916400; doi:10.1371/journal.ppat.1003911)
Supplement: Table S3 — Relative Infectivity of cell lines used. Numbers indicate the fold difference in viral input, relative to Vero E6 cells, required to achieve ∼30% infectivity in the indicated cells lines. (PDF) [file ppat.1003911.s013.pdf]

Table S3: Relative virus inputs for cell lines used

	rVSV-ANDV	VSV-(HTNV)	VSV-(G)
Vero E6	1	1	1
A549	10	5	2
CHO-K1	12	8	2
293T	1	5	1
HAP1	1	6.5	1

Values are normalized to virus input required to achieve ~30% infection in Vero E6 cells. Numbers reported are the fold increase in viral input needed to achieve similar infection levels in the indicated cell line.
